# TerrANTALife 1.0 Biodiversity data checklist of known Antarctic terrestrial and freshwater life forms

**DOI:** 10.3897/BDJ.12.e106199

**Published:** 2024-02-01

**Authors:** Luis R. Pertierra, Gilda Varliero, Andrés Barbosa, Elisabeth M. Biersma, Peter Convey, Steven L. Chown, Don Cowan, Asunción De Los Rios, Pablo Escribano-Alvarez, Diego Fontaneto, Ceridwen Fraser, Mathew Harris, Kevin Hughes, Huw Griffiths, Peter le Roux, Xiaoyue P. Liu, Heather Lynch, Roksana Majewska, Pablo A. Martinez, Marco Molina-Montenegro, Miguel A. Olalla-Tarraga, Lloyd Peck, Antonio Quesada, Cristina Ronquillo, Yan Ropert-Coudert, Leopoldo Sancho, Aleks Terauds, Juliana Vianna, Annick Wilmotte, Joaquín Hortal, Michelle Greve

**Affiliations:** 1 Department of Plant and Soil Sciences, University of Pretoria, Pretoria, South Africa Department of Plant and Soil Sciences, University of Pretoria Pretoria South Africa; 2 Millennium Institute of Biodiversity of Antarctic and Subantarctic Ecosystems (BASE), Santiago, Chile Millennium Institute of Biodiversity of Antarctic and Subantarctic Ecosystems (BASE) Santiago Chile; 3 Centre for Microbial Ecology and Genomics, University of Pretoria, Pretoria, South Africa Centre for Microbial Ecology and Genomics, University of Pretoria Pretoria South Africa; 4 Swiss Federal Institute for Forest, Snow and Landscape Research WSL, Birmensdorf, Switzerland Swiss Federal Institute for Forest, Snow and Landscape Research WSL Birmensdorf Switzerland; 5 Departamento de Ecología Evolutiva, Museo Nacional de Ciencias Naturales, CSIC, Madrid, Spain Departamento de Ecología Evolutiva, Museo Nacional de Ciencias Naturales, CSIC Madrid Spain; 6 Natural History Museum of Denmark, University of Copenhagen, Copenhagen, Denmark Natural History Museum of Denmark, University of Copenhagen Copenhagen Denmark; 7 British Antarctic Survey, Cambridge, United Kingdom British Antarctic Survey Cambridge United Kingdom; 8 Securing Antarctica's Environmental Future, Monash University, Victoria 3800, Melbourne, Australia Securing Antarctica's Environmental Future, Monash University, Victoria 3800 Melbourne Australia; 9 Departamento de Biogeoquimica y Ecologia Microbiana, Museo Nacional de Ciencias Naturales, CSIC, Madrid, Spain Departamento de Biogeoquimica y Ecologia Microbiana, Museo Nacional de Ciencias Naturales, CSIC Madrid Spain; 10 Departamento de Biología, Geología, Física y Química Inorgánica, Universidad Rey Juan Carlos, Mostoles, Spain Departamento de Biología, Geología, Física y Química Inorgánica, Universidad Rey Juan Carlos Mostoles Spain; 11 Water Research Institute, National Research Council of Italy, Verbania Pallanza, Italy Water Research Institute, National Research Council of Italy Verbania Pallanza Italy; 12 Department of Marine Science, University of Otago, Dunedin, New Zealand Department of Marine Science, University of Otago Dunedin New Zealand; 13 Britsh Antarctic Survey, Cambridge, United Kingdom Britsh Antarctic Survey Cambridge United Kingdom; 14 Department of Ecology and Evolution, Stony Brook University, New York, United States of America Department of Ecology and Evolution, Stony Brook University New York United States of America; 15 Faculty of Biosciences and Aquaculture, Nord University, Bodø, Norway Faculty of Biosciences and Aquaculture, Nord University Bodø Norway; 16 Unit for Environmental Sciences and Management, North-West University,, Potchefstroom, South Africa Unit for Environmental Sciences and Management, North-West University, Potchefstroom South Africa; 17 Laboratório de Pesquisa Integrativa em Biodiversidade (PIBi-Lab), Depto de Biologia, Universidade Federal de Sergipe, Aracaju, Brazil Laboratório de Pesquisa Integrativa em Biodiversidade (PIBi-Lab), Depto de Biologia, Universidade Federal de Sergipe Aracaju Brazil; 18 Instituto de Ciencias Biológicas, Universidad de Talca, Talca, Chile Instituto de Ciencias Biológicas, Universidad de Talca Talca Chile; 19 Centro de Investigación en Estudios Avanzados del Maule (CIEAM), Universidad Católica del Maule, Talca, Chile Centro de Investigación en Estudios Avanzados del Maule (CIEAM), Universidad Católica del Maule Talca Chile; 20 1Departmento de Biología, Geología, Física y Química Inorgánica, Universidad Rey Juan Carlos, Mostoles, Spain 1Departmento de Biología, Geología, Física y Química Inorgánica, Universidad Rey Juan Carlos Mostoles Spain; 21 Departamento de Biología, Universidad Autónoma de Madrid, Madrid, Spain Departamento de Biología, Universidad Autónoma de Madrid Madrid Spain; 22 Dept. de Biogeografía y Cambio Global, Museo Nacional de Ciencias Naturales, CSIC, Madrid, Spain Dept. de Biogeografía y Cambio Global, Museo Nacional de Ciencias Naturales, CSIC Madrid Spain; 23 Centre d'Etudes Biologiques de Chizé, , La Rochelle Université, Villiers-en-Bois, France Centre d'Etudes Biologiques de Chizé, , La Rochelle Université Villiers-en-Bois France; 24 Dept. de Biologia Vegetal II, Universidad Complutense de Madrid, Madrid, Spain Dept. de Biologia Vegetal II, Universidad Complutense de Madrid Madrid Spain; 25 Australian Antarctic Division, Department of Climate Change, Energy, the Environment and Water, Kingston, Australia Australian Antarctic Division, Department of Climate Change, Energy, the Environment and Water Kingston Australia; 26 Millennium Institute Center for Genome Regulation, Universidad Catolica de Chile, Santiago, Chile Millennium Institute Center for Genome Regulation, Universidad Catolica de Chile Santiago Chile; 27 InBios Research Unit, University of Liege, Liege, Belgium InBios Research Unit, University of Liege Liege Belgium; 28 Dept. of Plant and Soil Sciences, University of Pretoria, Pretoria, South Africa Dept. of Plant and Soil Sciences, University of Pretoria Pretoria South Africa

**Keywords:** Antarctica, biodiversity, polar fauna, polar flora, polar microorganisms, species inventories

## Abstract

**Background:**

Incomplete species inventories for Antarctica represent a key challenge for comprehensive ecological research and conservation in the region. Additionally, data required to understand population dynamics, rates of evolution, spatial ranges, functional traits, physiological tolerances and species interactions, all of which are fundamental to disentangle the different functional elements of Antarctic biodiversity, are mostly missing. However, much of the fauna, flora and microbiota in the emerged ice-free land of the continent have an uncertain presence and/or unresolved status, with entire biodiversity compendia of prokaryotic groups (e.g. bacteria) being missing. All the available biodiversity information requires consolidation, cross-validation, re-assessment and steady systematic inclusion in order to create a robust catalogue of biodiversity for the continent.

**New information:**

We compiled, completed and revised eukaryotic species inventories present in terrestrial and freshwater ecosystems in Antarctica in a new living database: terrANTALife (version 1.0). The database includes the first integration in a compendium for many groups of eukaryotic microorganisms. We also introduce a first catalogue of amplicon sequence variants (ASVs) of prokaryotic biodiversity. Available compendia and literature to date were searched for Antarctic terrestrial and freshwater species, integrated, taxonomically harmonised and curated by experts to create comprehensive checklists of Antarctic organisms. The final inventories comprises 470 animal species (including vertebrates, free-living invertebrates and parasites), 306 plants (including all Viridiplantae: embryophytes and green algae), 997 fungal species and 434 protists (sensu lato). We also provide a first account for many groups of microorganisms, including non-lichenised fungi and multiple groups of eukaryotic unicellular species (Stramenophila, Alveolata and Rhizaria (SAR), Chromists and Amoeba), jointly referred to as "protists". In addition, we identify 1753 bacterial (obtained from 348117 ASVs) and 34 archaeal genera (from 1848 ASVs), as well as, at least, 14 virus families. We formulate a basic tree of life in Antarctica with the main lineages listed in the region and their “known-accepted-species” numbers.

## Introduction

Antarctic terrestrial and freshwater diversity is richer and more complex than had long been thought (Convey and Stevens 2007), yet much of it remains poorly described (Chown et al. 2015). This unique biodiversity provides numerous ecosystem services to humankind, amongst them remarkable scientific insight (Pertierra et al. 2021). Even though available species inventories are still incomplete and unrefined, they offer invaluable insights on the structural biodiversity of the continent (Wauchope et al. 2019). Yet the existing biodiversity knowledge gaps affects our understanding and strategic protection of Antarctic ecosystems (Shaw et al. 2014, Hughes et al. 2015). Previous studies have compiled initial records of eukaryotic diversity from various sources serving to elucidate general biogeographical patterns (Terauds et al. (2012), Terauds and Lee (2016). However, in many instances, listed ‘species’ still lack taxonomic resolution and validation, while some groups are missing entirely (Overmann et al. 2019). The ongoing lack of systematic coverage of Antarctic diversity provides a continuing challenge to the adequate study and protection of life in the continent, increasingly threatened by progressive global change disturbances in the region (Siegert et al. 2019). Complete species inventories are critical for the comprehensive investigations of the prevailing biological patterns and processes in the region (Peat et al. 2007, Convey et al. 2014). Robust species inventories are also essential to designate and maintain effective protected area networks (Margules and Pressey 2000). Importantly, Antarctica is facing human-induced rapid environmental change that is likely to severely impact the marine and terrestrial life of the continent (Convey and Peck 2019, Lee et al. 2022). This makes more complete species inventories especially relevant for strategic conservation as often little is known about the spatial reservoirs of Antarctic biodiversity (Convey et al. 2020). Furthermore, systematic checklists allow for having all Antarctic biodiversity components minimally acknowledged and precautionarily preserved within Antarctic Specially Protected Areas under both the type locality criterion and other criteria of the Protocol on Environmental Protection to the Antarctic Treaty (Phillips et al. 2022).

The harsh and remote conditions of the Antarctic environment and the often cryptic nature of Antarctic biodiversity makes characterising its biota difficult (Carapelli et al. 2020). Nonetheless, imbued by the spirit of exploration for more than two centuries, Antarctic scientists have made steady progress in describing the species and communities of the continent (Leihy et al. 2019, Leihy et al. 2020). However, the singular status of the continent makes it challenging to overcome data fragmentation issues (Conix et al. 2021, Lien et al. 2021). As a result of all these factors, the degree to which different taxa have been characterised varies. The heterogeneous nature of survey and classification techniques used to explore the various forms of Antarctic biodiversity makes it difficult to create standardised data repositories for multi-taxa ecological applications. Survey efforts and classification methods vary widely between taxonomic and functional groups and have also changed over time.

Amongst eukaryotic groups, biological science in Antarctica has achieved different levels of progression in species discovery and taxonomic characterisation, with disparities similar to global paucity trends, but often also exacerbated by the regional logistic challenges of surveying the more cryptic groups in a harsh and remote continent. In the case of large marine breeding vertebrates, records of observations or locations of breeding colonies are considered to be legitimate survey records. Pathogenic invertebrates and microorganisms of vertebrate species in Antarctica are surveyed by tagging and/or collating samples from blood and other host tissues in dedicated studies either requiring capture and release or dead host specimens and kept in glass slide fixations (Wilson 1997, Houstin et al. 2021). For free-living soil microinvertebrates, whole organisms are typically collected using extraction methods such as flotation, pitfall traps or soil cores and Tullgren or Berlese funnels (Treonis et al. 1999, Enríquez et al. 2019) and, after identification, are stored in institutional or museum collections (although subsequent maintenance and curation of collections varies). Aquatic microinvertebrates, algae and heterotrophic protists (SAR-complex and Amoeba) are filtered from waterbody samples and preserved in fixations (Maturana et al. 2022). Raw diatom material is either preserved in ethanol or air-dried and stored or acid-cleaned, preserved in ethanol and stored in institutional, museum or private collections (Majewska et al. 2016). Plants and lichens are typically collected by hand-picking and lodged in herbaria (Peat 1998, Peat et al. 2007). Fungi, algae and bacteria can be incorporated in national or institutional culture collections. Macroscopic fruiting bodies (mushrooms/toadstools) are also sometimes included in herbaria, whereas microscopic organisms are kept in slides (Peat 1998). Altogether the history of biological science studies in the continent provides an array of collections and compendia of variable size across eukaryotic groups from where to identify biodiversity knowledge gaps.

Next-generation sequencing (NGS) has provided a means of detecting and characterising Antarctic microorganisms. Microorganism identifications, in particular, are now often proposed through next-generation sequencing of environmental samples (e.g. Fraser et al. (2017), Barnes and Turner (2015), Dragone et al. (2021). Surveys are commonly conducted through the sequencing of genes characterised by both universal and variable regions, such as the 16S rRNA genes for prokaryotic organisms and ITS, COI and 18S rRNA genes for eukaryotic microorganisms (Tringe and Hugenholtz 2008, Qiang-long et al. 2014, Banos et al. 2018). Microbial identification by NGS of these genes is far from perfect as it relies on a polymerase chain reaction (PCR) approach which is known to be biased towards diverse microorganisms, depends on the used primers and the amplified gene regions and on online taxonomic databases which are not comprehensive and representative of all organisms (Hayashi Sant’Anna et al. 2019, Emerson et al. 2022). Many examples exist of Antarctic microorganisms isolated and cultured from the environment (e.g. Franzmann et al. (1988), Baraniecki et al. (2002)), but it is widely recognised that only a minority of representatives of these groups can be brought into culture and that culture-dependent approaches can only access a (sometimes tiny) proportion of the diversity present.

Much scientific research and conservation nowadays depends on the availability and reliability of the supporting local and regional species inventories (Whittaker et al. 2005). For instance, the delivery of adequate systematic conservation planning relies on having comprehensive species inventories and evolutionary hierarchies, also of rare or vulnerable species (Margules and Pressey 2000). In addition, the examination of evolutionary, macroecological and/or biogeographical patterns and processes is often severely hampered by species knowledge gaps and biases (Hortal et al. 2015). As conservation efforts often aim to maintain high biodiversity levels, accurate diversity estimates are necessary to adequately measure diversity variation and to determine whether conservation policies should be implemented (Meyer et al. 2015). Not knowing which species are present leads to misguided protection priorities, miscalculated representation levels and low confidence in identifying potential impacts to local biodiversity as part of environmental assessments (Thomson et al. 2021).

The Antarctic Biodiversity Information Facility (AntaBIF) platform within the Global Biodiversity Information Facility (GBIF) represents a centralised digital meta-repository of Antarctic biodiversity datasets that facilitates the integration of Antarctic biodiversity data (Gan et al. 2019). However, much of the existing biodiversity data cover marine ecosystem biodiversity. Only some terrestrial, freshwater and intertidal species are present, with knowledge gaps affecting their protection (Hawes et al. 2023). Even though field specimen classification remains challenging for many of these cryptic groups, this issue is being surmounted as new computer-aided taxonomic identification techniques unfold (Saucède et al. 2020) and the growing DNA blasting libraries further support consolidation of regional inventories (Elshishka et al. 2023, Collins et al. 2023). Such fast developments require broader data centralisation efforts to keep the growing inventories regularly updated, integrated and accessible (Gan et al. 2019). Currently, the most regularly updated compendium of Antarctic terrestrial diversity is the Scientific Committee on Antarctic Research (SCAR) ‘Antarctic Biodiversity Database’ (ABD) which compiles terrestrial species records, covering over 2,207 reported eukaryotic terrestrial and freshwater entities from the Antarctic (Wauchope et al. 2019). However, much of the fauna, flora and microbiota included in the ABD has uncertain presence data and/or an unresolved status and additional biodiversity compendia (e.g. for bacteria) are missing. To improve the current situation, all the available Antarctic terrestrial biodiversity information requires consolidation, cross-validation, re-assessment and steady systematic inclusion in order to create a ‘catalogue of life’ for the continent. In response to this priority need (Koerich et al. 2023), here we present the new terrANTAlife biodiversity checklist, as a holistic ‘catalogue of life’ compendium. The dataset is in an interchangeable file DarwinCore format with integration to GBIF and has an extended information toolset lodged in the parent directory at the CSIC digital open access server. Moreover, this dataset builds on previous efforts by the Antarctic community and allows for future integration with other repositories in order to achieve an internationally-coordinated, fully complete biodiversity inventory for the Antarctic Region. The terrANTAlife compendium helps fill current knowledge gaps in existing Antarctica biodiversity inventories by providing a FAIR set of complete, comprehensive, inclusive and novel biodiversity checklist toolsets that are intended to guide both future holistic biodiversity research studies in the region, as well as to provide information for strategic conservation policies (Antarctic Treaty Secretariat 2000, Hobern et al. 2021).

## General description

### Purpose

In this report, we generate a comprehensive revised list of the terrestrial and freshwater species and lineages present in the Antarctic continent. Specifically, we aim to address the lack of coverage of microbiota, while also revising the existing knowledge on fauna and flora. Where available, we retrieve lists of species (or higher taxonomic levels when specified) cited in existing repositories and/or classical compendia and update these with the latest published work. We also cross-validate their acceptance status against the latest nomenclature available from global taxonomical facilities. We thereby provide a renewed tool with which to evaluate the biodiversity knowledge of Antarctica.

### Additional information

The current dataset is presented as a freely-available resource that operates as a living repository of Antarctic species, aiming to contribute with biodiversity data consolidation in regional and global information facilities, such as ANTABif, whilst already being integrated in GBIF (http://gbif-chile.mma.gob.cl/ipt/resource?r=terrantalife_eukariota). Furthermore, the datasets are formulated to allow for regular updates and corrections resulting from ongoing and new research finding.

## Project description

### Title

ANTALIFE 1.0 Biodiversity data checklist of all Antarctic terrestrial and freshwater lifeforms

### Personnel

**Conceptual Design** LR Pertierra, G Varliero, M Greve, J Hortal, SL Chown

**Parataxonomists** (data listings): LR Pertierra (all groups), P EscribanoAlvarez (invertebrates), M Harris (fungi), P Liu (protists), G Varliero (procaryotes), KA Hughes (fungi and procaryotes), H Lynch (vertebrates), L Peck (algae and crustaceans), A Terauds (all groups).

**Curators** (taxonomic revision): L Sancho & A DeLosRios (fungi & lichen), M MolinaMontengro & P LeRoux (vascular plants), E Biersma (bryophytes), P Convey, D Fontaneto, A Barbosa, H Griffiths (invertebrates), R Majewska, C Fraser & A Wilmotte (algae, protist and diatoms), J Vianna & Y Roupert-Coudet (vertebrates), A Quesada & D Cowan (procaryotes).

### Study area description

The continent of Antarctica. All emerged lands and water bodies south of -60 Latitude.

### Design description

Antarctic biodiversity data (species inventories) compilation for all terrestrial and freshwater living organisms through expert curation.

## Sampling methods

### Sampling description

**Figure 1.**
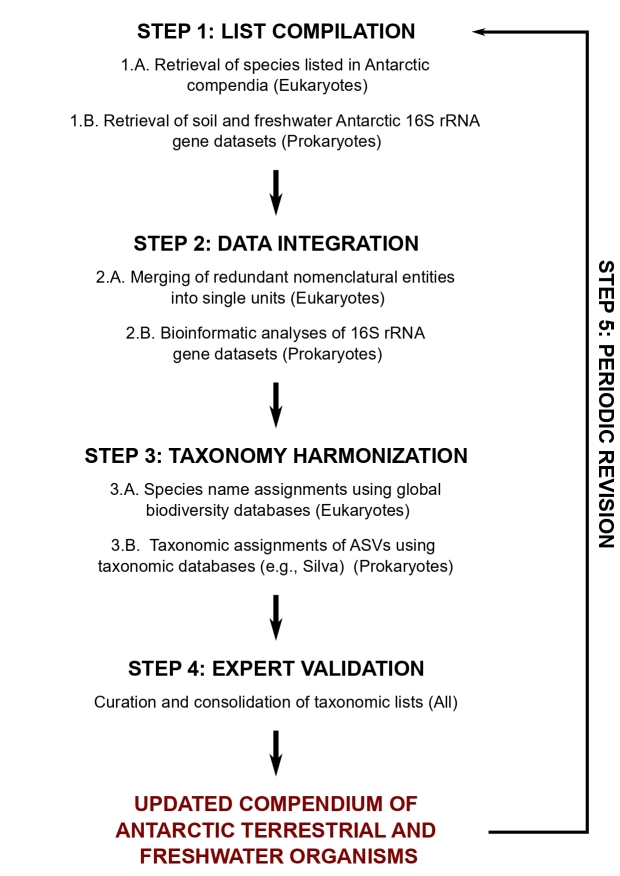
Biodiversity checklist compilation protocol.

A set of rules and guidance was created to generate a robust standardised checklist that would meet the conditions of representation and inclusion, systematic harmonisation, proofing, transparency and dynamism. Based on these rules in order to generate the revised biodiversity checklist across groups, we constructed a generalised stepwise validation procedure tailored for eukaryotes and prokaryotes (see Fig. 1).

**List compilation.** To generate an initial compendium of Antarctic biodiversity, we retrieved all the species listed in the existing major diversity compendia for the continent. We started with the Wauchope et al. (2019) list of species as a backbone and added further taxa. A list of parasitic animal species records was included from the Barbosa and Palacios (2009) compendium. In the case of Rotifera, a pre-existing checklist of species was available in the AntaBIF database to GBIF (https://www.gbif.org/dataset/b109fc97-b7b8-4432-a89a-5eaaadeee431). Additional lists of bryophytes were obtained from the Ochyra et al. (2008) (moss) and Bednarek-Ochyra et al. (2000) (liverworth) compendia. A complementary list of lichens was retrieved from Øvstedal and Lewis Smith (2001)'s compendium. A list of non-lichenised fungal species was retrieved from the BAS fungal database (Bridge et al. 2010). The SAR group redefines the previous terminology of the paraphyletic group of “protists” and includes the SAR subclades of Stramenopiles, Alveolata and Rhizaria, along with Amoeobozoa and others (Ruggiero et al. 2015). The latter are not related to SAR and we merely used them here in line with current convention. The works of Adams et al. 2006 and Thompson et al. 2019 were taken as the source compendium of SAR species. The initial diatom species list was retrieved from the recent and comprehensive biogeographical study of Verleyen et al. (2021) which includes known freshwater species; sub-Antarctic species listed in that study were excluded. The list was supplemented with information on terrestrial diatom species recorded from Antarctica in the 20^th^ and 21^st^centuries and taxonomic papers describing freshwater diatom taxa published after 2021. In the absence of published compendia, additional lists of species representing other SAR groups and Amoebae were retrieved directly from the relevant literature. To obtain a list of bacterial and archaeal genera found in soil and freshwater Antarctic environments, we re-analysed data from publicly available 16S rRNA gene datasets (PROKARYOTIC STUDY SOURCE LIST data set). Eukaryotic viruses and bacteriophages remain very poorly described in Antarctica. We, therefore, only identify the main lineages reported from the region in recent literature.

Lastly, for groups without an initial species list, we retrieved candidate species from review works and/or Antarctic literature searches. Firstly, participant curator co-authors screened seminal book compendia. Next, a complementary screening of literature was performed for inclusion/updating from recent discoveries. The species inventories were augmented with recently published findings (updated to October 2023). Antarctic literature searches were done in Web of Science repository using basic terms per group with the following syntax e.g. “Taxon*” AND Antarctic*”. The complete list of queries can be seen in Suppl. material 1. Participant parataxonomist co-authors were assigned subset lists of works around the taxon/taxa queried according to their main expertise. From the extracted literature, we first excluded all studies that were not specifically conducted in the continent of Antarctica. Second, we browsed through the titles and abstracts of literature to identify works that could contain previously unreported species. For instance, species inventories for some groups of flora and fauna have been recently reviewed with regards to the existence of specific type locality data (Phillips et al. 2022) providing the opportunity to grow the dataset with previous omissions and/or new species detections. Additional annotations were made with regards of the content of the manuscripts, but these were not subsequently analysed here. A particular focus was given to the publications of the last five years. The possibility exists that further new material exists, but the intention of this exercise is to be dynamically active and that further revisions will be made to the living inventories over time.

### Quality control

**Data integration.** Participant parataxonomists looked at the eukaryotic species datasets to detect and merge duplicates and species synonyms between lists and assign their accepted nomenclature. Aggregation of synonym species identities (current status) was based on global biodiversity facilities. We used GBIF (https://www.gbif.org/) as the primary source to link up names and synonyms to formally accepted species worldwide and to retrieve the year of first description of the species and taxonomic authorities. For certain taxa, other sources were used to standardise and check nomenclature, namely ITIS (https://www.itis.gov/), Micobank (Robert et al. 2013; https://www.mycobank.org/), NCBI (Schoch et al. 2020; https://www.ncbi.nlm.nih.gov/taxonomy), AlgaeBase (Guiry and Guiry 2023, https://www.algaebase.org/), WORMS (WORMS Editorial Board 2023, https://www.marinespecies.org/) and IUCN (https://www.iucn.org/). Entries with still unresolved species-level status were left separated as doubtful.

To generate the bacterial list, online repositories were mined for 16S rRNA gene Antarctic soil and freshwater datasets of Illumina amplicon sequences (Tytgat et al. 2016, Zhang et al. 2020, Almela et al. 2021, Mashamaite et al. 2023, Dragone et al. 2021, Borsetto et al. 2019, Solon et al. 2021, Ortiz et al. 2021, Meier et al. 2019, Ji et al. 2022, Lutz et al. 2019, Varliero et al. 2024, Staebe et al. 2019, Severgnini et al. 2021, Picazo et al. 2021, Ramoneda et al. 2021, Weisleitner et al. 2019, Saxton et al. 2021, Sommers et al. 2017, Fernández et al. 2022, Webster-Brown et al. 2015, Kleinteich et al. 2017, Achberger et al. 2016). All retrieved reads were analysed to obtain consistent ASVs and genus-level taxonomic classification. All sequences were analysed using the dada2 pipeline (Callahan et al. 2016) in the R environment (R Core Team 2021), with the use of the libraries phyloseq (McMurdie and Holmes 2013), Biostrings (Pagès et al. 2021) and decontam (Davis et al. 2018). Archaeal information was obtained using the same datasets and pipeline as for the bacteria. However, the low archaeal diversity (especially when compared to the bacterial diversity, in terms of retrieved ASVs) might be inflated because most prokaryotic universal primers show low specificity for Archaea (Bahram et al. 2018). Bacterial and archaeal organisms were reported as ASVs (Callahan et al. 2017) obtained from NGS studies conducted on soil and freshwater environmental samples. We report taxonomic information for bacterial and archaeal organisms at the genus-level for reference, from which prokaryotic genera could potentially be found in the Antarctic environment. However, it should be kept in mind that this taxonomic classification is subject to limitations intrinsic to bioinformatics pipelines and that not all the species present in a reported genus can necessarily be found in Antarctic.

**Taxonomical harmonisation.** Most recent systematic procedures are nowadays directly in the handbooks of online facilities. Nonetheless, the following monograph served as an inspiration for our guiding principles (Thiele et al. 2021, Pyle et al. 2021) by attending taxonomic completion and revision with defined scopes (Antarctica, all biodiversity groups, all available records since the first exploration of the continent), a set granularity (species for eucaryotes and ASVs for procaryotes), defined rules of arbitration for curation (see expert validation) and transparency in the form of version tracking. The remaining challenge presented in this dataset arises from the lack of attribution of confidence levels to each reclassification, a question that is intended to be amended in future versions. Eukaryotic species curation was based on the Catalogue of Life approach (Hobern et al. 2021). Identified synonyms were corrected and brought up to date. Organisms of doubtful identity that were excluded during the biodiversity list generation are listed separately in the datasets as they represent species that are yet to be accepted in global repositories or are lacking taxonomic resolution. As each taxonomic subgroup faced its own challenges, we handled these with a backbone treatment, whilst still incorporating subtle differences where required. For instance, the Species 2000 - Catalogue of Life systematics (Roskov et al. 2020) was used as the backbone for most taxa, but was replaced by AlgalBase and MicoBank for algae and fungi, respectively, as these are considered more reliable/updated (Guiry 2012). Species listed that appeared as synonyms were replaced by the currently accepted names, with their original syntax retained in notes. A list of Antarctic species that could not be found as accepted or synonyms were classified as doubtful; these could indicate additional species that they are yet to be formally accepted.

For bacterial and archaeal organisms, ASVs were annotated using the SILVA database v. 138 (Quast et al. 2012). Taxonomy is here represented as all genera obtained for all the re-analysed datasets. Thefore the genus list collates all the genera present in at least one of the datasets.

**Expert validation.** Animal data were revised by nine Antarctic faunal experts. Plant/algae data were revised by five Antarctic flora experts. Lichen/fungi data were revised by four fungal experts. Bacteria/Archaea data was revised by five bacterial experts.

## Geographic coverage

### Description

Terrestrial taxonomic lists were limited to the emerged ice and land areas of Antarctic Treaty Governance south of 60 Degrees Latitude, including the linked Scotia Arc South Shetland Islands and South Orkney Islands, but excluding the maritime Antarctic South Sandwich Islands and Bouvetøya (which lie north of the Antarctic Treaty area) and the sub-Antarctic islands. Based on the admittedly imperfect biodiversity assessments currently available, 16 Antarctic Conservation Biogeographic Regions (ACBRs) have been identified (Terauds and Lee 2016, Terauds et al. 2012) and taken as reference. These bioregions were validated independently by Verleyen et al. (2021).

Antarctic terrestrial and freshwater biodiversity primarily comprise a remarkably diverse microcosm of small and microscopic organisms, with very few examples of marine breeding vertebrates or terrestrial vascular plants. This biodiversity primarily occurs, but is not limited to, the patchy and rare ice-free areas of the continent (Convey 2017), which are typically divided in Antarctic ecoregions (ACBRs) (Terauds and Lee 2016). Although much of the Antarctic biodiversity is restricted to the ice-free areas, microbial systems are also present in the iced environments that make up more than 99.5% of the continent (Burton-Johnson et al. 2016, Chown et al. 2022). Thus, Antarctic terrestrial and freshwater ecosystems include an extreme range of macro- and microhabitats (Convey et al. 2014, Chown et al. 2022). Maritime-influenced ice-free habitats are small and disperse, but frequent in the coasts of the continent and offshore islands. They include beaches, valleys and slopes exposed by retreating glaciers under the influence of the sea, creating a mosaic of cryptogam-dominated fellfields, moss carpets and peats overlying poorly-developed soils, with drainage systems of often ephemeral streams and shallow ponds and deeper lakes. Such ecosystems are best developed on the Antarctic Peninsula and Scotia Arc archipelagos (maritime Antarctic), but are also present in the more limited ice-free areas around the continental Antarctic coast and inland. Much of the Antarctic diversity is widely present here, including groups like phaenoragam plants that are absent elsewhere in the continent. The characteristic ‘Dry valleys’ of the continent are concentrated in parts of the Transantarctic Mountains (Chan et al. 2013), but are also found elsewhere on the continent, such as on Alexander Island in the southern Antarctic Peninsula (Convey and Smith 1997). Groups that do occur in these areas include very small numbers of mosses, lichens, microarthropods (mites and springtails) and microinvertebrates (nematodes, tardigrades, rotifers) (Adams et al. 2006), with overall diversity being dominated by soil microbial groups which form biological soil crusts and also develop in hypo- and endolithic habitats (Cary et al. 2010, de los Ríos et al. 2014), along with benthic algal/cyanobacterial mats in freshwater bodies (Velázquez et al. 2016). Nunataks are mountain summits and ridges that protrude from the surrounding Antarctic ice sheet and host low diversity communities of lichens (and some mosses to around latitude 76°S) and invertebrates (e.g. Convey and McInnes (2005)). Ice and snow environments can also support some life; their communities are dominated by a diversity of microorganisms, such as bacteria and snow algae and micro-invertebrates, such as tardigrades (Anesio et al. 2017, Davey et al. 2019). Lastly, sub-glacial ecosystems, also present at the ground/ice interface under the continental ice sheets, represent one of the last unexplored ecosystems, where microbial species are known to thrive (Christner et al. 2014, Mikucki et al. 2016). In this study, we incorporate all the living organisms described to occur, to our knowledge, in these habitats.

### Coordinates

-90o and -60o Latitude; -180o and 180o Longitude.

## Taxonomic coverage

### Description

All Antarctic living organisms and viral groups reported for the continent were included here. It is essential to be inclusive of all taxonomic groups regardless of the knowledge gaps and different methodologies that are intrinsic to their study (Shaw et al. 2014). Therefore, eukaryotes are reported at the species level and prokaryotes at the ASV level. To comprehensively represent Antarctic environmental prokaryotes, we deem an ASV approach more accurate compared to a species-level approach because it does not rely on any taxonomic sequence databases. The latter are, in fact, highly incomplete for environmental prokaryotes (Hayashi Sant’Anna et al. 2019). Further, ASV information was obtained by compiling several environmental datasets which represented different regions of the 16S rRNA gene and, therefore, no accurate classification down to species-level was possible. Together with ASV information, we also report taxonomy at the genus-level for prokaryotic organisms.

We included Antarctic terrestrial and freshwater living organisms and sorted them under the seven kingdom classification of Ruggiero et al. (2015). Additionally, viral particles were also listed separately. Breeding marine vertebrates (seals and nesting seabirds) and their endo- and ectoparasites were included under terrestrial diversity as they spend part of their life cycle on land and represent an important source of physical disturbance and nutrient enrichment to terrestrial ecosystems (Bokhorst et al. 2019). Stranded marine algae in the supralittoral zone and marine intertidal species were excluded as they spend their life cycle in seawater. Snow algae were included. Ice-sheet life, such as picoplankton, was excluded. Microorganisms from soil and freshwater, such as lakes and glacial environments (e.g. cryoconites and subglacial lakes) were included. No airborne microorganisms were listed, but future revisions would possibly benefit from these).

### Taxa included

**Table:** 

Rank	Scientific Name	Common Name
kingdom	Animalia (Metazoa)	Pluricellular animals
kingdom	Plantae	Plants
kingdom	Fungi	Fungi and lichen
kingdom	Bacteria	Bacteria
kingdom	Archaea	Archaeae
kingdom	Amoebozoa	Amoebas
kingdom	SAR/Protist	Protists / protozoans in sensu lato
form	Virus	Virus and bacteriophages

## Temporal coverage

### Notes

Version 1.0 of ANTABASE contains species records up to and including December 2022. To provide a living database, it is anticipated that checklists will be updated over time, with post-launch curation following the successful and pragmatic approach of GBIF. The checklists will be updated regularly with new versions, provided, ideally, on an annual basis, post publication. To this end, members of the polar research community will be regularly consulted via social media and events asking for new contributions and updates to include. It is proposed that the updates shall be coordinated by a committee comprised of members of the Antarctic research community under the auspices of SCAR, with approval of proposed updates confirmed following appropriate peer-review. Each version of the dataset will be made available to enable any changes, errors and/or sources to be traced back.

Antarctica is governed through consensus by the Consultative Parties to the Antarctic Treaty, with decision-making occurring at the now annual Antarctic Treaty Consultative Meeting (ATCM). The Committee for Environmental Protection (CEP) provides advice to the ATCM on issues relating to the protection of the Antarctic Environment. Through its Five-Year Work Plan (available at https://www.ats.aq/e/committee.html), the CEP has identified ‘Biodiversity knowledge’ as essential to provide information for this work. It is anticipated that this biodiversity dataset will be presented as a policy paper to the CEP as the best available science regarding biodiversity knowledge of the Antarctic terrestrial and freshwater environment. The information may assist the ATCM and CEP in its decision-making, including the conservation of species and habitats through designation of Specially Protected Species and protected areas and the delivery of the Environmental Impact Assessment (EIA) process. The database is also a contribution to the SCAR Scientific Research Programme ‘Integrated Science to Inform Antarctic and Southern Ocean Conservation’ (Ant-ICON) Theme 1 ‘Current state and future projections of Antarctic Southern Ocean and sub-Antarctic systems, species and functions‘.

## Usage licence

### Usage licence

Оpen Data Commons Open Database License (ODbL)

## Data resources

### Data package title

terrANTALIFE Antarctic terrestrial and freshwater species inventories

### Resource link

https://digital.csic.es/handle/10261/307449

### Number of data sets

6

### Data set 1.

#### Data set name

terraANTALIFE_eukariotic_v1.0

#### Data format

Text (CSV UTF-8)

#### Download URL

https://digital.csic.es/handle/10261/307449

#### Description

Inventory of eukaryotic species in terrestrial and freshwater ecosystems of Antarctica. Version 1.0 (08.11.2023). Taxonomic levels follow DarwinCore descriptions.

**Data set 1.** 

Column label	Column description
taxonID	Internal identification number.
modified	Date of latest modification.
usageKey	Identificator number in GBIF.
kingdom	The full scientific name of the kingdom in which the taxon is classified.
phylum	The full scientific name of the phylum in which the taxon is classified.
class	The full scientific name of the class in which the taxon is classified.
family	The full scientific name of the family in which the taxon is classified.
genus	The full scientific name of the genus in which the taxon is classified.
TaxonRankGBIF	Scientific name of the lowest classification rank accepted by GBIF with the author of description (in most cases, corresponds to the scientific name of the species).
ScientificName	Accepted scientific name given by the prevailing repository used as reference. In GBIF extension, this column title is renamed as verbatimScientificName.
taxonRank	Level of the lowest classification rank accepted by GBIF.
Confidence	Certainty in the taxon re-assignation made by GBIF.
synonym	Synonym status (TRUE/FALSE) according to GBIF. Scientific names allocated by other prevailing repositories that differ from GIF are listed as TRUE until either GBIF updates their status (by accepting them as accepted species) or the other repository ceases the claim.
namePublishedInYear	Year of first discovery and description of the species by the original author/s (anywhere on Earth).
namePublishedInYearGBIF	Year of first record of the species in Antarctica lodged in GBIF.
namePublishedInYearsSACS	Earlier year of presence in Antarctica taken for the Species Accumulation curves. Taken from either the YearofDiscovery (for endemic species) or the YearGBIF (for global species).
nameAccordingtoMycoBank	AcceptedSpecies name in MycoBank repository. Prioritised field for AcceptedSpecies assignation in fungi.
scientificNameAuthorshipMycoBank	Authorship recognised in Mycobank.
taxonRemarks	Annotation of alternative name synonyms locally given to the taxon.
antarcticBibliographicCitation	Publication indicating the latest presence of the species in the continent, where possible source citation replaces central checklists to specific reporting works.
namePublishedIn	Publication of the new species description, limited to species found in Antarctica. It can involve endemic and non-endemic species first found there.
establishmentMeans	Automatic classification of the biogeographical distribution of the species, based from the global range of occurrences in GBIF.
expertlifeformRemarks	Attributed lifeform for the species. Between free-living, symbiont or parasitic species (also where known). Completeness largely biased towards fungi and invertebrates.
verbatimScientificNameAuthorship	Authorship given by the prevailing source material used as reference.

### Data set 2.

#### Data set name

terraANTALIFE_eukaryota_v1.0_GBIF_Extension

#### Download URL

http://gbif-chile.mma.gob.cl/ipt/resource?r=terrantalife_eukariota

#### Description

Data integration of 'terraANTALIFE_eukaryotic_v1.0' to GBIF extension checklist. This is a shortened version with the basic biodiversity information integrated in GBIF.

**Data set 2.** 

Column label	Column description
All columns	Same as 'terraANTALIFE_eukaryotic_v1.0'.

### Data set 3.

#### Data set name

terrANTALIFE_prokaryotes_genus_list_v1

#### Download URL

https://digital.csic.es/handle/10261/307449

#### Description

Inventory of prokaryotic genera in terrestrial and freshwater ecosystems of Antarctica. Version 1.0 (08.11.2023). Taxonomic levels follow DarwinCore descriptions.

**Data set 3.** 

Column label	Column description
kingdom	The full scientific name of the kingdom in which the taxon is classified.
phylum	The full scientific name of the phylum or division in which the taxon is classified.
class	The full scientific name of the class in which the taxon is classified.
order	The full scientific name of the order in which the taxon is classified.
family	The full scientific name of the family in which the taxon is classified.
genus	The full scientific name of the genus in which the taxon is classified.

### Data set 4.

#### Data set name

ANTALIFE_prokaryotes_asv_v1.fasta

#### Data format

FASTA

#### Download URL

https://digital.csic.es/handle/10261/307449

#### Description

Fasta file reporting all ASV sequences assigned to the kingdoms Archaea and Bacteria. Taxonomy associated to each ASV sequence is reported in the header as domain, phylum, class, order, family, genus, species. When an ASV was unclassified at a particular taxonomic level, "NA" is reported instead.

**Data set 4.** 

Column label	Column description
DNA headers and sequences	Headers and ASV sequences reported in fasta format.

### Data set 5.

#### Data set name

terraANTALIFE_prokaryota_v1.0_GBIF_Extension. This is a shortened version with the basic biodiversity information integrated in GBIF.

#### Download URL

http://gbif-chile.mma.gob.cl/ipt/resource?r=terrantalife_prokariota

**Data set 5.** 

Column label	Column description
All columns	Same as 'terraANTALIFE_prokaryota_v1.0'.

### Data set 6.

#### Data set name

terrANTALIFEProkaryotic study_source_list_v1.01.csv

#### Data format

Text (CSV UTF-8)

#### Download URL

https://digital.csic.es/handle/10261/307449

#### Description

Specifics of all datasets collated to create list of ASVs and genera for prokaryotes.

**Data set 6.** 

Column label	Column description
Repository accession	Accession code to access dataset in public repositories.
Paper	Peer-reviewed paper first reporting a dataset.
DOI	Peer-reviewed paper DOI.
Sample collection year	Year of sample collection.
Location collection	Site of sample recovery.
Environmental medium	Type of sample material.
DNA extraction kit	Supplier of sample processing kit for DNA extraction (by provider).
Illumina technology	Illumina technology used to process samples.
Primer set	Primer set used to amplify 16S rRNA genes.
16S rRNA gene variable region	Amplified 16S rRNA gene variable region.
Number of samples	Number of samples in each dataset.
Prokaryotic kingdom	Prokaryotic kingdom.
Number of phyla	Number of phyla associated with a dataset.
Number of classes	Number of classes associated with a dataset.
Number of orders	Number of orders associated with a dataset.
Number of families	Number of families associated with a dataset.
Number of genera	Number of genera associated with a dataset.
Number of ASVs	Number of ASVs associated with a dataset.
Percentage of ASVs assigned at genus-level	Percentage of ASVs assigned at genus-level.

## Additional information

### Results and discussion

**1. Numbers of identifiable Antarctic biodiversity units and recent biodiversity findings**

A total of 1870 eukaryotic species with a currently-accepted status and a total of 349,966 prokaryotic ASVs were obtained from our collations. Thirty of the 149 green algae listed as pseudo-species in Wauchope et al. (2019) were accepted by GBIF. This indicates that much of the chlorophyte taxonomy is still unconfirmed. A total of 468 non-lichenised fungi listed by Bridge et al. (2010) were also recognised in MicoBank. We report bacterial (Datasets 2-3 of the Data Package) and archaeal (Datasets 4-5 of the Data package) diversity by presenting the number of genera and ASVs found for the Antarctic soil and freshwater datasets.

ANIMALIA (METAZOA) KINGDOM 470 species total in version 1.0.

Breeding vertebrates. Twenty-six species in version 1.0. Amongst marine vertebrates utilising the continent, no new species descriptions have been proposed in more than a century. However, the taxonomy of a few species of macrofauna has been revisited as a result of advances in molecular biological methodologies that can be applied in describing their phylogenetic relationships (e.g. Techow et al. (2009), Vianna et al. (2020)).

Arthropods. A total of 189 species in version 1.0. In contrast to their marine counterparts in the Southern Ocean, arthropods are not a dominant component of Antarctic freshwater fauna, often occurring in low abundances and diversity. Our latest knowledge of the distribution of non-marine freshwater arthropods comes from Díaz et al. (2019). Only three new freshwater species have been named since the start of the 21^st^ century (*Diacyclopskaupi*, *D.walkeri* and *D.joycei*) by Karanovic et al. (2014). Arthropods are, however, prominent in soil ecosystems. The species inventories and phylogeography amongst terrestrial arthropod invertebrates, such as springtails (e.g. Carapelli et al. (2020)), midges and mites (e.g. Brunetti et al. (2021), Collins et al. (2023)) continues to be explored with sometimes highly challenging reassessments (Stevens and D'Haese 2016). In addition, new species of invertebrate ectoparasites continue to be reported (Barbosa and Palacios 2009, Montero et al. 2016).

Non-arthropod invertebrates. A total of 254 species in version 1.0. Amongst other animal phyla, there has been a recent surge in descriptions of new species of terrestrial and freshwater non-arthropod invertebrates, including both Antarctic regional or short-range endemic species, again encouraged by the application of advanced molecular phylogenetic and phylogeographic techniques. This also includes multiple instances of the identification of species-level (or greater) evolutionary divergences within species considered to date as single species and of the presence of cryptic speciation, with multiple such species yet to be formally described. Latest examples include tardigrades (e.g. Short et al. (2022)) and nematodes (e.g. Bostrom et al. (2011)), but not rotifers, for which species seem to be broadly distributed in Antarctica (Cakil et al. 2021). In addition, new species of invertebrate gastroparasites continue to be reported (Montero et al. 2016). Overall, new systematic re-classification techniques, based on molecular markers, are being explored in diverse microscopic animal groups, such as tardigrades (Vecchi et al. 2016). Inventories have recently been compiled and subsequently revised and updated for some invertebrate groups, such as rotifers (Iakovenko et al. 2015, Garlasché et al. 2019).

PLANTAE KINGDOM. A total of 306 species in version 1.0.

Embryophyte plants. A total of 154 species in version 1.0. Angiosperms and bryophytes represent one of the best known groups in Antarctica. Only two vascular plant species occur in Antarctica, with both having wider sub-Antarctic and South American distributions; therefore, no new species descriptions have been made for quite some time. Contemporary diversity research is now examining their precise evolutionary identities (Biersma et al. 2020). This is also applicable to bryophytes, which are relatively well known, though their taxonomical relationships are still being unveiled (Biersma et al. 2018, Camara et al. 2019).

Green algae. A total of 152 species in version 1.0. New species of free-living green algae are expected to be described as we continue to explore the continent. In turn, recent studies examine green algae diversity as photobionts in lichens (Ruprecht et al. 2012, Garrido‐Benavent and Pérez‐Ortega 2017) and as free-living algal forms in snow and water systems (Davey et al. 2019)

FUNGI KINGDOM. A total of 997 species in version 1.0.

Fungi. A total of 871 Ascomycota species, 95 Basidiomycota and 41 Zygomycota and others (in version 1.0). New species of lichen-forming fungi are still being described (e.g. Garrido-Benavent et al. 2016) with currently over 500 known entities identifiable in our dataset), but also current research also focuses on disentangling phylogenetic relationships (e.g. Lagostina et al. (2017)). Non-lichenised fungi represent a more cryptic group that continues to be explored, but a substantial number (> 500) of globally-distributed species have been recorded from Antarctica (Bridge et al. 2010).

AMOEBA, PROTOZOA (Ciliata and Flagellata) AND SAR/CHROMISTA KINGDOM COMPLEXES. 434 SPECIES.

SAR. A total of 418 species in version 1.0. Amongst the SAR supercomplex, biogeographical research in diatoms (Stramenophyles, Ochrophyta, Bacillariophyceae) is a growing discipline (Verleyen et al. 2021). Here, we detected 287 diatom species in total. In addition, five Xanthophyta species were also isolated from biogeographical studies (Broady 1996). However, studies on the diversity of heterotrophic protists (Alveolata, Rhizaria) are a bit more stagnant, but see pioneer efforts (Thompson et al. 2019). Their current known diversity to our knowledge involves A-R 130 species plus one Chrysophyceae.

Protozoa. Seven species in version 1.0. Flagellata and Ciliophora are also inconsistently recorded, with systematic observations in but a few regions (Adams et al. 2006). Additionally, new species continue to be described (Park et al. 2020). Only seven Antarctic protozoan species were found here, representing one of the largest Antarctic biodiversity knowledge gaps.

Oomycota (Chromista). Four species in version 1.0. A total of four Oomycota species were listed in Bridge et al. (2010).

Amoeba (Sarcodina). One accepted species in version 1.0. One single Amoeba species (Platyamoeba stenopodia) remains described (Adams et al. 2006). However, additional protozoans with *incerta* status are likely to be placed in the group once their classification is resolved.

BACTERIA AND ARCHAEA KINGDOMS. A total of 349,966 prokaryotic ASVs in version 1.0.

Prokaryotes. Whereas we know that microbial communities are adapted to live in diverse Antarctic challenging habitats (Dragone et al. 2021), these communities are widely unexplored. Environmental surveys from soil and freshwater Antarctic habitats routinely find unidentified microorganisms (Bowman 2018). Our re-analysis of a selection of publicly available 16S rRNA amplicon datasets resulted in only 37% and 82% of taxonomically assigned ASVs at the genus level (see PROKARYOTIC STUDY SOURCE LIST dataset). This high portion of unknown microorganisms highlights the knowledge gap in microbial communities and the consequent gaps in taxonomic reference databases (Bowman 2018). However, as both sampling and sequencing technology advance, new microbial habitats are explored, new sequencing datasets are obtained and new microorganisms are characterised (Taş et al. 2021). In addition to amplicon studies, whole shotgun metagenome is also allowing advances in microbial taxonomy explorations with the reconstruction of complete metagenome assembled genomes (MAGs), allowing the discovery of full genomes without the use of culturing techniques (Tully et al. 2018, Yang et al. 2021). Metagenomic and metatranscriptomic data are, therefore, pivotal for the exploration of microbial diversity and metabolic functions, in particular, in life-challenging environments, such as Antarctica where cell isolation and culture in laboratory settings are rarely possible (Bodor et al. 2020).

VIRAL ENTITIES. Fourteen families in version 1.0.

Viruses. We have a very coarse notion of viral diversity, which is thought to be remarkably high (Lopez-Bueno et al. 2009, Zablocki et al. 2014). Pathogenic viruses in Antarctic wildlife have received increased attention in recent years. Wildlife pathogens can be transported to Antarctic via migratory species (e.g. Antarctic fur seals, Arctic terns or skuas) arriving from areas where the pathogen already exists or through human activity (Barbosa et al. 2021). Direct or serological evidence has been detected in Antarctic wildlife for viral diseases including Infectious Bursal Disease Virus, Newcastle Disease Virus, Petrelpox Virus, Sealpox Virus, Canine Distemper Virus, Phocine Distemper Virus and Phocine Herpes virus 1 (Barbosa and Palacios 2009, Kerry and Riddle 2009, Grimaldi et al. 2014). Recent concerns regarding the risk of transmission of SARS-CoV-2 from human to Antarctic wildlife have resulted in the production of mitigation measures, although there remain no reports of COVID-19 in Antarctic wildlife to date.

**2. Examination of the Antarctic Tree of Life: phylodiversity elements in the continent**

**Figure 2.**
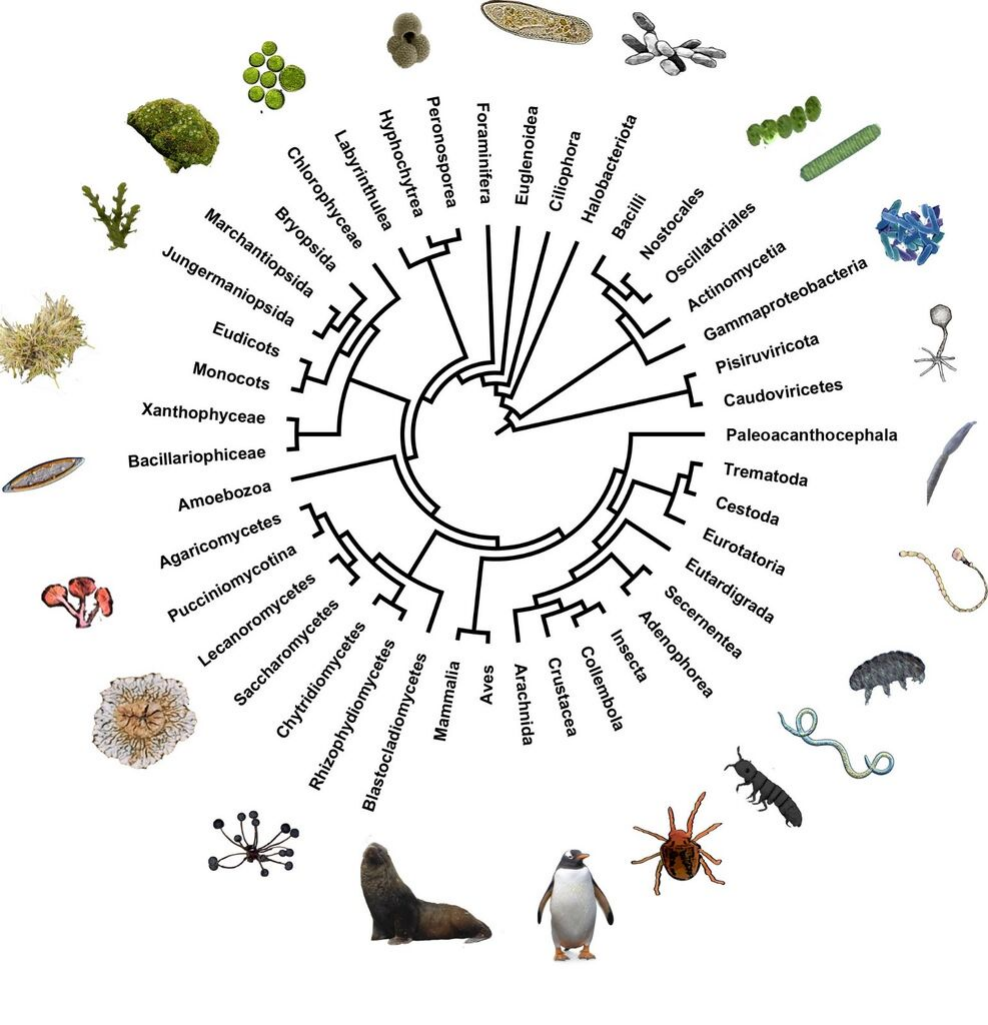
Basic Antarctic Tree of Life. Some lesser animal phyla with less than five species total are not visually depicted (e.g. Annelida).

Basic phylogenetic relationships between taxa were established from the Tree of Life project (http://tolweb.org/tree/). A Tree of Life for Antarctic diversity was created using the major realms, kingdoms, phyla and classes present in the region (Fig. 2). Due to the widely differing levels of diversity within each clade, we used only one representative taxon for each (once these diverged too broadly at the root of the trees to be depicted in full). This representative was typically drawn from the most recurrent higher taxa amongst each clade in our biodiversity list. Thus, only one group of viruses and phages were selected, respectively. For SAR and bacteria, only some representative supergroup lineages were taken. In turn, for fauna and flora (plant, fungi and algae), most groups were represented at the level of class.

Overall, the Antarctic continent hosts over 400 species from at least seven animal phyla (see Fig. 2). Considering the general diversity of the region, most terrestrial phyla are present even in this “barren” continent. Amongst freshwater annelids, only a few *Lumbricillus*/*Marionina* species (five in total) are reported to be present in the continent's stream waters (Rodriguez and Rico 2008), but a non-native entrytraeid worm *Christenseniablocki* is also locally present in Signy Island (Dózsa-Farkas and Convey 1997, Hughes et al. 2015). No terrestrial or freshwater molluscs are known from the region but, if the intertidal zone is included, some marine species are present (Jossart et al. 2023). No non-marine bryozoans have been reported from the region. Additionally, no freshwater fish, reptiles (*sensu lato*) or amphibians are present. Insect diversity is remarkably low, with representatives of only one free-living order present (two species of Diptera), along with another two parasitic (Psocodea and Siphonaptera).

Plant diversity is also high in the continent, yet the full extent of Viridiplantae diversity is still uncertain. In the case of embryophytes, the most remarkable contemporary absence is that of ferns, along with the very low diversity of vascular plants (two species, one native Caryophyllaceae and another Poaceae), but significantly covering both monocots and eucots presence in the continent. No gimnosperms are present, but they were widely present at some point in the paleohistory of the continent, as observed from fossil records. A remarkable diversity of bryophytes is found, with all three major divisions present (mosses, hornworts and liverworts). Both insects and vascular plants become more diverse in the sub-Antarctic islands.

Amongst fungi, compared with global patterns, Antarctic diversity is relatively lower amongst Basidiomycota and higher in Ascomycota, but all major groups are present at the division level, including some mushroom-forming species of Basidiomycota and Ascomycota. SAR and others (formerly described as “protists”) are also diverse and include representatives of most recognised groups in the region. However, major surveying and knowledge gaps still exist and knowledge of diversity remains far from complete.

The unique combination of phylogenetic diversity in the Antarctic is threatened by the arrival and establishment of non-native species (Duffy et al. 2017). Futhermore, pre-established alien species in Antarctica present singular invasive traits, with the risk of altering the functional behaviour of the ecosytems (Pertierra et al. 2022). Fortunately, at present, these remain limited for the most part to soil arthropods (Acari, Insecta and Collembolla).

**3. Challenges to constitute more accurate biodiversity compendia**

Continued development of modern molecular and integrated taxonomic methodologies is paramount for further improving the assessment of species identity and representative inclusion. Detailed and extensive taxonomic revisions are required for most groups, also founded on wider mobilisation to local studies and expertise generating frequently updated species list repositories. The ongoing need for biodiversity data compilation and integration into repositories, such as GBIF and AntaBIF, is currently a major challenge for ecological research, at the same time being particularly relevant for strategic conservation planning. New primary survey data are required to generate accurate knowledge of diversity in many areas of the continent that, even today, remain unvisited by group specialists. The role of taxonomists is especially relevant for filling gaps and, therefore, the training of new taxonomists is essential. The very diverse range of poorly-understood microbiological groups that are bundled under the term ‘microbial biodiversity’, namely viruses, bacteria, archaea, microscopic fungi and algae and protozoans, represent a major research gap of the region.

**Eukaryotes**. No high-level faunal groups were absent from Antarctic inventories prior to the preparation of this compendium. However, the availability and application of new molecular and integrated taxonomic approaches are leading to an upsurge in new species descriptions, especially for non-arthropod micro-invertebrates and Acari and Collembola (Collins et al. 2023). Most embryophyte groups are also well studied, with robust compendia available that can be updated with recent reconsideration of a small number of species identities of mosses (Ochyra et al. 2008) and liverworths (Bednarek-Ochyra et al. 2000). In contrast, eukaryotic green algae continue to be a highly uncertain group where the known species diversity is likely to represent a fraction of the true total present. Lichen-forming fungal diversity is similarly well studied (Øvstedal and Lewis Smith 2001), with a broad spectrum of non-lichenised fungal species records in the region also available (Bridge et al. 2010). However, much fungal diversity remains unknown, with increasing evidence from eDNA and other molecular analyses supporting the presence of substantial unknown diversity within this group (Rosa et al. 2020).

**Prokaryotes.** Considerable advances in understanding microbial diversity in specific Antarctic regions have been made in the last decade through the application of newly-available sequencing and metagenomic technologies (Taş et al. 2021) and it is clear that the potential of these is only just beginning to be tapped. These are allowing exploration of the microbial diversity previously unexplored Antarctic regions and habitats, such as Dronning Maud Land nunataks (Staebe et al. 2019) and the Transantarctic Mountains (Dragone et al. 2021). However, while the potential of these approaches is clear, important limitations remain, in particular, relating to the large-scale incompleteness of available sequence databases against which to assign sequence identities and the lack of ability to confirm the presence or function of viable biota. Many ‘new’ ASVs are typically found in newly-analysed samples, suggesting (as with the fungi above) that Antarctic microbial diversity is far from well-characterised. Effort has also been devoted in recent years to describe microbial distribution patterns in relation to habitat conditions, bioclimatic variables and geographical distances (Chong et al. 2015, Chown et al. 2015, Almela et al. 2021). Better characterisation of these patterns, both at micro- and macro- scales, will provide valuable information on microbial diversity and how better to protect and conserve it. Again, increased sampling effort and the development of considerably more comprehensive microbial taxonomy online databases are required to facilitate improved microbial diversity characterisation. Lastly, further research is also required to enhance understanding of disease prevalence and transmission in Antarctic wildlife and the associated effects of increasing human activity and climate change in the region.

### Conclusions

The Antarctic is one of the most remote and harsh regions on Earth; therefore, Antarctic diversity remains challenging to document comprehensively. Conversely, the relatively low diversity of most taxonomic groups in the region and moderate total number of phyla make the preparation of semi-complete compendia of diversity more feasible. Moreover, Antarctica also represents one of the regions of strongest international collaboration and multi-taxa research, thus offering a unique opportunity to have a complete picture of the existing biodiversity for a region of Earth. The dataset presented here provides a considerable improvement in Antarctic biodiversity knowledge, both in terms of species identities in several groups and in wider group representation, with the first recognition of several previously-unlisted groups. Characterising Antarctic diversity represents a difficult, but achievable challenge. The development of comprehensive biodiversity databases is required to enable the increased recognition and representation of “lesser” taxonomic groups in both biological sciences research and conservation assessments. We strongly advocate the examination and identification of biodiversity knowledge gaps and the compendium presented here gives a powerful tool to assist in such assessments in terms of species coverage, spatial distribution and temporal change.

## Supplementary Material

82D9EF40-9934-546F-B56E-A9DEB91625EA 10.3897/BDJ.12.e106199.suppl1 Supplementary material 1 Bibliographic search queries in Web of Science on Antarctic biodiversity findings

Data type search code Brief description List of search queries per group made in Web of Science for additional Antarctic species detection in recent and historical literature on the biodiversity of the continent. File: oo_846121.txt https://binary.pensoft.net/file/846121 All

## References

[B11068839] Achberger Amanda M., Christner Brent C., Michaud Alexander B., Priscu John C., Skidmore Mark L., Vick-Majors Trista J. (2016). Microbial Community Structure of Subglacial Lake Whillans, West Antarctica. Frontiers in Microbiology.

[B9043510] Adams Byron J., Bardgett Richard D., Ayres Edward, Wall Diana H., Aislabie Jackie, Bamforth Stuart, Bargagli Roberto, Cary Craig, Cavacini Paolo, Connell Laurie, Convey Peter, Fell Jack W., Frati Francesco, Hogg Ian D., Newsham Kevin K., O’Donnell Anthony, Russell Nicholas, Seppelt Rodney D., Stevens Mark I. (2006). Diversity and distribution of Victoria Land biota. Soil Biology and Biochemistry.

[B9141255] Almela Pablo, Justel Ana, Quesada Antonio (2021). Heterogeneity of microbial communities in soils from the Antarctic Peninsula region. Frontiers in Microbiology.

[B9140862] Anesio Alexandre M., Lutz Stefanie, Chrismas Nathan A. M., Benning Liane G. (2017). The microbiome of glaciers and ice sheets. npj Biofilms and Microbiomes.

[B9635166] Secretariat Antarctic Treaty (2000). Guidelines for implementation of the Framework for Protected Areas set forth in Article 3, Annex V of the Environmental Protocol. Annexed to Resolution 1. Available at: https://documents.ats.aq/recatt/Att081_e.pdf. https://documents.ats.aq/recatt/Att081_e.pdf.

[B9140487] Bahram Mohammad, Anslan Sten, Hildebrand Falk, Bork Peer, Tedersoo Leho (2018). Newly designed 16S rRNA metabarcoding primers amplify diverse and novel archaeal taxa from the environment. Environmental Microbiology Reports.

[B9140226] Banos Stefanos, Lentendu Guillaume, Kopf Anna, Wubet Tesfaye, Glöckner Frank Oliver, Reich Marlis (2018). A comprehensive fungi-specific 18S rRNA gene sequence primer toolkit suited for diverse research issues and sequencing platforms. BMC Microbiology.

[B9140355] Baraniecki C. A., Aislabie J., Foght J. M. (2002). Characterization of *Sphingomonas* sp. Ant 17, an aromatic hydrocarbon-degrading Bacterium isolated from Antarctic soil. Microbial Ecology.

[B9043378] Barbosa Andrés, Palacios María José (2009). Health of Antarctic birds: a review of their parasites, pathogens and diseases. Polar Biology.

[B9635612] Barbosa Andrés, Varsani Arvind, Morandini Virginia, Grimaldi Wray, Vanstreels Ralph E T, Diaz Julia I, Boulinier Thierry, Dewar Meagan, González-Acuña Daniel, Gray Rachael, McMahon Clive R, Miller Gary, Power Michelle, Gamble Amandine, Wille Michelle (2021). Risk assessment of SARS-CoV-2 in Antarctic wildlife.. The Science of the total environment.

[B9140186] Barnes Matthew A., Turner Cameron R. (2015). The ecology of environmental DNA and implications for conservation genetics. Conservation Genetics.

[B9635149] Bednarek-Ochyra H., Váňa J., Ochyra R., Lewis Smith R. I. (2000). The liverwort flora of Antarctica.

[B9714357] Biersma EM, Jackson JA, Stech M, Griffiths H, Linse K, Convey P (2018). Molecular data suggest long-term in Situ Antarctic persistence within Antarctica's most speciose plant genus, Schistidium. Frontiers in Ecology and Evolution.

[B9714515] Biersma E. M., Torres-Díaz C., Newsham K. K., Vidal M. A., Ballesteros G., Figueroa C. C., Goodall-Copestake W. P., Leppe M. A., Cuba-Díaz M., Valladares M. A., Pertierra L. R., Convey P., Acuña-Rodríguez I. S., Collado G. A., Molina-Montenegro M. A. (2020). Multiple late-Pleistocene colonisation events of the Antarctic pearlwort *Colobanthusquitensis* (Caryophyllaceae) reveal the recent arrival of native Antarctic vascular flora. Journal of Biogeography.

[B9141185] Bodor Attila, Bounedjoum Naila, Vincze György Erik, Erdeiné Kis Ágnes, Laczi Krisztián, Bende Gábor, Szilágyi Árpád, Kovács Tamás, Perei Katalin, Rákhely Gábor (2020). Challenges of unculturable bacteria: environmental perspectives. Reviews in Environmental Science and Bio/Technology.

[B9635202] Bokhorst Stef, Convey Peter, Aerts Rien (2019). Nitrogen inputs by marine vertebrates drive abundance and richness in Antarctic terrestrial ecosystems.. Current Biology : CB.

[B11068586] Borsetto Chiara, Amos Gregory C. A., da Rocha Ulisses Nunes, Mitchell Alex L., Finn Robert D., Laidi Rabah Forar, Vallin Carlos, Pearce David A., Newsham Kevin K., Wellington Elizabeth M. H. (2019). Microbial community drivers of PK/NRP gene diversity in selected global soils. Microbiome.

[B9714644] Bostrom S., Holovachov S., Nadler S. A. (2011). Description of *Scottnemalindsayae* Timm, 1971 (Rhabditida: Cephalobidae) from Taylor Valley, Antarctica and its phylogenetic relationship. Polar Biology.

[B9141070] Bowman Jeff S. (2018). Identification of microbial dark matter in Antarctic environments. Frontiers in Microbiology.

[B9634963] Bridge P, Spooner B. M., Roberts P. List of non-lichenized fungi from the Antarctic region. https://data.bas.ac.uk/full-record.php?id=GB/NERC/BAS/PDC/00235.

[B9635157] Broady P. (1996). Diversity distribution and dispersal of Antarctic terrestrial algae. Biodiversity and Conservation.

[B9714592] Brunetti C., Siepel H., Convey P., Fanciulli P. P., Nardi F., Carapelli A. (2021). Overlooked species diversity and distribution in the Antarctic mite genus *Stereotydeus*. Diversity.

[B9713171] Burton-Johnson A., Black M., Fretwell P. T., Kaluza-Gilbert J. (2016). An automated methodology for differentiating rock from snow, clouds and sea in Antarctica from Landsat 8 imagery: a new rock outcrop map and area estimation for the entire Antarctic continent. The Cryosphere.

[B9142962] Cakil Zeyneb Vildan, Garlasché Giuseppe, Iakovenko Nataliia, Di Cesare Andrea, Eckert Ester M., Guidetti Roberto, Hamdan Lina, Janko Karel, Lukashanets Dzmitry, Rebecchi Lorena, Schiaparelli Stefano, Sforzi Tommaso, Kašparová Eva Štefková, Velasco‐Castrillón Alejandro, Walsh Elizabeth J., Fontaneto Diego (2021). Comparative phylogeography reveals consistently shallow genetic diversity in a mitochondrial marker in Antarctic bdelloid rotifers. Journal of Biogeography.

[B9140432] Callahan Benjamin J, McMurdie Paul J, Rosen Michael J, Han Andrew W, Johnson Amy Jo A, Holmes Susan P (2016). DADA2: High-resolution sample inference from Illumina amplicon data.. Nature Methods.

[B9140412] Callahan Benjamin J, McMurdie Paul J, Holmes Susan P (2017). Exact sequence variants should replace operational taxonomic units in marker gene data analysis. bioRxiv.

[B9714501] Camara P., Soares A., Henriques D., Peralta D., Bordin J., Carvalho-Silva M., Stech M. (2019). New insights into the species diversity of *Bartramia* Hedw. (Bryophyta) in Antarctica. Antarctic Science.

[B9139034] Carapelli Antonio, Greenslade Penelope, Nardi Francesco, Leo Chiara, Convey Peter, Frati Francesco, Fanciulli Pietro Paolo (2020). Evidence for cryptic diversity in the “Pan-Antarctic” Springtail *Frieseaantarctica* and the description of two new species. Insects.

[B9140871] Cary S. Craig, McDonald Ian R., Barrett John E., Cowan Don A. (2010). On the rocks: the microbiology of Antarctic Dry Valley soils. Nature Reviews Microbiology.

[B9713240] Chan Yuki, Nostrand J. D.Van, Zhou J., Pointing S. B., Farrell R. L. (2013). Functional ecology of an Antarctic Dry Valley. Proceedings of the National Academy of Sciences.

[B9141245] Chong Chun-Wie, Pearce David A., Convey Peter (2015). Emerging spatial patterns in Antarctic prokaryotes. Frontiers in Microbiology.

[B9042901] Chown Steven L., Clarke Andrew, Fraser Ceridwen I., Cary S. Craig, Moon Katherine L., McGeoch Melodie A. (2015). The changing form of Antarctic biodiversity. Nature.

[B9713180] Chown S. L., Leihy R. I., Naish T. R., Brooks C. M., Convey P., Henley B. J., Mackintosh A. N., Phillips L. M., Kennicutt M. C., Grant S. M. (2022). Antarctic climate change and the environment: A decadal synopsis and recommendations for action..

[B9635517] Christner Brent C, Priscu John C, Achberger Amanda M, Barbante Carlo, Carter Sasha P, Christianson Knut, Michaud Alexander B, Mikucki Jill A, Mitchell Andrew C, Skidmore Mark L (2014). A microbial ecosystem beneath the West Antarctic ice sheet. Nature.

[B10820713] Collins Gemma E., Young Monica R., Convey Peter, Chown Steven L., Cary S. Craig, Adams Byron J., Wall Diana H., Hogg Ian D. (2023). Biogeography and Genetic Diversity of Terrestrial Mites in the Ross Sea Region, Antarctica. Genes.

[B9143019] Conix Stijn, Garnett Stephen T., Thiele Kevin R., Christidis Les, van Dijk Peter Paul, Bánki Olaf S., Barik Saroj K., Buckeridge John S., Costello Mark J., Hobern Donald, Kirk Paul M., Lien Aaron, Nikolaeva Svetlana, Pyle Richard L., Thomson Scott A., Zhang Zhi-Qiang, Zachos Frank E. (2021). Towards a global list of accepted species III. Independence and stakeholder inclusion. Organisms Diversity & Evolution.

[B9713254] Convey P., Smith R. I.L. (1997). The terrestrial arthropod fauna and its habitats in northern Marguerite Bay and Alexander Island, maritime Antarctic. Antarctic Science.

[B9713291] Convey Pete, McInnes Sandra J. (2005). Exceptional Tardigrade-dominated ecosystems in Ellsworth Land, Antarctica. Ecology.

[B9127629] Convey Peter, Stevens Mark I. (2007). Antarctic Biodiversity. Science.

[B9127285] Convey Peter, Chown Steven L., Clarke Andrew, Barnes David K. A., Bokhorst Stef, Cummings Vonda, Ducklow Hugh W., Frati Francesco, Green T. G. Allan, Gordon Shulamit, Griffiths Huw J., Howard-Williams Clive, Huiskes Ad H. L., Laybourn-Parry Johanna, Lyons W. Berry, McMinn Andrew, Morley Simon A., Peck Lloyd S., Quesada Antonio, Robinson Sharon A., Schiaparelli Stefano, Wall Diana H. (2014). The spatial structure of Antarctic biodiversity. Ecological Monographs.

[B9635387] Convey Peter, Levin Simon A (2017). Encyclopedia of biodiversity.

[B9635312] Convey Peter, Peck Lloyd S. (2019). Antarctic environmental change and biological responses. Science Advances.

[B9139134] Convey Peter, Biersma Elisabeth M., Casanova-Katny Angelica, Maturana Claudia S. (2020). Refuges of Antarctic diversity. Past Antarctica.

[B9043486] Davey Matthew P., Norman Louisa, Sterk Peter, Huete‐Ortega Maria, Bunbury Freddy, Loh Bradford Kin Wai, Stockton Sian, Peck Lloyd S., Convey Peter, Newsham Kevin K., Smith Alison G. (2019). Snow algae communities in Antarctica: metabolic and taxonomic composition. New Phytologist.

[B9140469] Davis Nicole M., Proctor Diana M., Holmes Susan P., Relman David A., Callahan Benjamin J. (2018). Simple statistical identification and removal of contaminant sequences in marker-gene and metagenomics data. Microbiome.

[B9140880] de los Ríos Asunción, Cary Craig, Cowan Don (2014). The spatial structures of hypolithic communities in the Dry Valleys of East Antarctica. Polar Biology.

[B9714622] Díaz Angie, Maturana Claudia S, Boyero Luz, De Los Ríos Escalante Patricio, Tonin Alan M, Correa-Araneda Francisco (2019). Spatial distribution of freshwater crustaceans in Antarctic and Subantarctic lakes.. Scientific Reports.

[B9635707] Dózsa-Farkas K., Convey P. (1997). *Christensenia*, a new terrestrial enchytraeid genus from Antarctica. Polar Biology.

[B9140195] Dragone Nicholas B., Diaz Melisa A., Hogg Ian D., Lyons W. Berry, Jackson W. Andrew, Wall Diana H., Adams Byron J., Fierer Noah (2021). Exploring the boundaries of microbial habitability in soil. Journal of Geophysical Research: Biogeosciences.

[B9143147] Duffy Grant A., Coetzee Bernard W. T., Latombe Guillaume, Akerman Alexander H., McGeoch Melodie A., Chown Steven L. (2017). Barriers to globally invasive species are weakening across the Antarctic. Diversity and Distributions.

[B10820704] Elshishka Milka, Mladenov Aleksandar, Lazarova Stela, Peneva Vlada (2023). Terrestrial nematodes from the Maritime Antarctic. Biodiversity Data Journal.

[B9140248] Emerson Brent, Borges Paulo, Cardoso Pedro, Convey Peter, deWaard Jeremy, Economo Evan, Gillespie Rosemary, Kennedy Susan, Krehenwinkel Henrik, Meier Rudolf, Roderick George, Strasberg Dominique, Thébaud Christophe, Traveset Anna, Creedy Thomas, Meramveliotakis Emmanouil, Noguerales Victor, Overcast Isaac, Morlon Hélène, Papadopoulou​ Anna, Vogler Alfried, Arribas Paula, Andujar Carmelo (2022). Collective and harmonised high throughput barcoding of insular arthropod biodiversity: toward a Genomic Observatories Network for islands. Authorea, Inc..

[B10816282] Enríquez Natalia, Pertierra Luis R., Tejedo Pablo, Benayas Javier, Greenslade Penelope, Luciáñez María José (2019). The importance of long-term surveys on species introductions in Maritime Antarctica: first detection of Ceratophysella succinea (Collembola: Hypogastruridae). Polar Biology.

[B11068785] Fernández Guillermo Cesar, Lecomte Karina, Vignoni Paula, Rueda Eliana Soto, Coria Silvia H., Lirio Juan M., Mlewski Estela Cecilia (2022). Prokaryotic diversity and biogeochemical characteristics of benthic microbial ecosystems from James Ross Archipelago (West Antarctica). Polar Biology.

[B9140342] Franzmann P. D., Stackebrandt E., Sanderson K., Volkman J. K., Cameron D. E., Stevenson P. L., Mcmeekin T. A., Burton H. R. (1988). *Halobacteriumlacusprofundi* sp. nov., a halophilic Bacterium isolated from Deep Lake, Antarctica. Systematic and Applied Microbiology.

[B9140208] Fraser Ceridwen I., Connell Laurie, Lee Charles K., Cary S. Craig (2017). Evidence of plant and animal communities at exposed and subglacial (cave) geothermal sites in Antarctica. Polar Biology.

[B10820728] Gan Yi-Ming, Sweetlove Maxime, Van de Putte Anton (2019). The Antarctic Biodiversity Portal, an Online Ecosystem for Linking, Integrating and Disseminating Antarctic Biodiversity Information. Biodiversity Information Science and Standards.

[B9142945] Garlasché Giuseppe, Karimullah Karimullah, Iakovenko Nataliia, Velasco-Castrillón Alejandro, Janko Karel, Guidetti Roberto, Rebecchi Lorena, Cecchetto Matteo, Schiaparelli Stefano, Jersabek Christian D., De Smet Willem H., Fontaneto Diego (2019). A data set on the distribution of Rotifera in Antarctica. Biogeographia – The Journal of Integrative Biogeography.

[B9635739] Garrido-Benavent I., Søchting U., de los Ríos Murillo A., Pérez-Ortega S. (2016). *Shackletoniacryodesertorum* (Teloschistaceae, Ascomycota), a new species from the McMurdo Dry Valleys (Antarctica) with notes on the biogeography of the genus *Shackletonia*. Mycological Progress.

[B9635773] Garrido‐Benavent Isaac, Pérez‐Ortega Sergio (2017). Past, present, and future research in bipolar lichen-forming fungi and their photobionts. American Journal of Botany.

[B9043446] Grimaldi Wray W., Seddon Phil J., Lyver Phil O’B., Nakagawa Shinichi, Tompkins Daniel M. (2014). Infectious diseases of Antarctic penguins: current status and future threats. Polar Biology.

[B9634805] Guiry Michael D (2012). How many species of algae are there?. Journal of Phycology.

[B9634797] Guiry M. D., Guiry G. M. AlgaeBase. World-wide electronic publication, National University of Ireland, Galway. https://www.algaebase.org/.

[B10820650] Hawes Ian, Howard-Williams Clive, Gilbert Neil, Hughes Kevin A., Convey Peter, Quesada Antonio (2023). The need for increased protection of Antarctica's inland waters. Antarctic Science.

[B9140331] Hayashi Sant’Anna Fernando, Bach Evelise, Porto Renan Z., Guella Felipe, Hayashi Sant’Anna Eduardo, Passaglia Luciane M. P. (2019). Genomic metrics made easy: what to do and where to go in the new era of bacterial taxonomy. Critical Reviews in Microbiology.

[B9143094] Hobern Donald, Barik Saroj K., Christidis Les, Stephen T. Garnett,, Kirk Paul, Orrell Thomas M., Pape Thomas, Pyle Richard L., Thiele Kevin R., Zachos Frank E., Bánki Olaf (2021). Towards a global list of accepted species VI: The Catalogue of Life checklist. Organisms Diversity & Evolution.

[B9043099] Hortal Joaquín, de Bello Francesco, Diniz-Filho José Alexandre F., Lewinsohn Thomas M., Lobo Jorge M., Ladle Richard J. (2015). Seven shortfalls that beset large-scale knowledge of biodiversity. Annual Review of Ecology, Evolution, and Systematics.

[B10820772] Houstin Aymeric, Zitterbart Daniel P., Winterl Alexander, Richter Sebastian, Planas-Bielsa Víctor, Chevallier Damien, Ancel André, Fournier Jérôme, Fabry Ben, Le Bohec Céline (2021). Biologging of emperor penguins – attachment techniques and associated deployment performance. bioRxiv.

[B9139062] Hughes Kevin A., Ireland Louise C., Convey Peter, Fleming Andrew H. (2015). Assessing the effectiveness of specially protected areas for conservation of Antarctica's botanical diversity. Conservation Biology.

[B9714535] Iakovenko Nataliia, Smykla Jerzy, Convey Peter, Kasparova Eva, Kozeretska Iryna, Trokhymets Vladlen, Dykyy Igor (2015). Antarctic bdelloid rotifers: diversity, endemism and evolution. Hydrobiologia.

[B11068679] Ji Mukan, Kong Weidong, Jia Hongzeng, Delgado-Baquerizo Manuel, Zhou Tianqi, Liu Xiaodong, Ferrari Belinda C., Malard Lucie, Liang Chao, Xue Kai, Makhalanyane Thulani P., Zhu Yong-Guan, Wang Yanfen, Pearce David A., Cowan Don (2022). Polar soils exhibit distinct patterns in microbial diversity and dominant phylotypes. Soil Biology and Biochemistry.

[B9634865] Jossart Quentin, Bauman David, Moreau Camille Ve, Saucède Thomas, Christiansen Henrik, Brasier Madeleine J, Convey Peter, Downey Rachel, Figuerola Blanca, Martin Patrick, Norenburg Jon, Rosenfeld Sebastian, Verheye Marie, Danis Bruno (2023). A pioneer morphological and genetic study of the intertidal fauna of the Gerlache Strait (Antarctic Peninsula).. Environmental Monitoring and Assessment.

[B9714612] Karanovic T., Gibson J., Hawes I., Andersen D., Stevens M. (2014). *Diacyclops* (Copepoda: Cyclopoida) in Continental Antarctica, including three new species. Antarctic Science.

[B9635667] Kerry Knowles R., Riddle Martin J. (2009). Health of Antarctic wildlife: A challenge for science and policy.

[B11068819] Kleinteich Julia, Hildebrand Falk, Bahram Mohammad, Voigt Anita Y., Wood Susanna A., Jungblut Anne D., Küpper Frithjof C., Quesada Antonio, Camacho Antonio, Pearce David A., Convey Peter, Vincent Warwick F., Zarfl Christiane, Bork Peer, Dietrich Daniel R. (2017). Pole-to-Pole Connections: Similarities between Arctic and Antarctic Microbiomes and Their Vulnerability to Environmental Change. Frontiers in Ecology and Evolution.

[B10820679] Koerich Gabrielle, Fraser Ceridwen I., Lee Charles K., Morgan Fraser J., Tonkin Jonathan D. (2023). Forecasting the future of life in Antarctica. Trends in Ecology & Evolution.

[B9635716] Lagostina Elisa, Dal Grande Francesco, Ott Sieglinde, Printzen Christian (2017). Fungus-specific SSR markers in the Antarctic lichens *Usneaantarctica* and *U.aurantiacoatra* (Parmeliaceae, Ascomycota). Applications in Plant Sciences.

[B9042912] Lee Jasmine R., Terauds Aleks, Carwardine Josie, Shaw Justine D., Fuller Richard A., Possingham Hugh P., Chown Steven L., Convey Peter, Gilbert Neil, Hughes Kevin A., McIvor Ewan, Robinson Sharon A., Ropert-Coudert Yan, Bergstrom Dana M., Biersma Elisabeth M., Christian Claire, Cowan Don A., Frenot Yves, Jenouvrier Stéphanie, Kelley Lisa, Lee Michael J., Lynch Heather J., Njåstad Birgit, Quesada Antonio, Roura Ricardo M., Shaw E. Ashley, Stanwell-Smith Damon, Tsujimoto Megumu, Wall Diana H., Wilmotte Annick, Chadès Iadine (2022). Threat management priorities for conserving Antarctic biodiversity. PLOS Biology.

[B9043078] Leihy Rachel I., Coetzee Bernard W. T., Morgan Fraser, Raymond Ben, Shaw Justine D., Terauds Aleks, Chown Steven L. (2019). Antarctica's wilderness has declined to the exclusion of biodiversity. bioRxiv.

[B9043065] Leihy Rachel I., Coetzee Bernard W. T., Morgan Fraser, Raymond Ben, Shaw Justine D., Terauds Aleks, Bastmeijer Kees, Chown Steven L. (2020). Antarctica’s wilderness fails to capture continent’s biodiversity. Nature.

[B9143041] Lien Aaron M., Conix Stijn, Zachos Frank E., Christidis Les, van Dijk Peter Paul, Bánki Olaf S., Barik Saroj K., Buckeridge John S., Costello Mark John, Hobern Donald, Montgomery Narelle, Nikolaeva Svetlana, Pyle Richard L., Thiele Kevin, Thomson Scott A., Zhang Zhi-Qiang, Garnett Stephen T. (2021). Towards a global list of accepted species IV: Overcoming fragmentation in the governance of taxonomic lists. Organisms Diversity & Evolution.

[B9714664] Lopez-Bueno A., Tamames J., Velazquez D., A Moya, A Quesada, Alcami A. (2009). High diversity of the viral community from an Antarctic lake. Science.

[B11068776] Lutz Stefanie, Ziolkowski Lori A., Benning Liane G. (2019). The Biodiversity and Geochemistry of Cryoconite Holes in Queen Maud Land, East Antarctica. Microorganisms.

[B10820809] Majewska Roksana, Convey Peter, De Stefano Mario (2016). Summer Epiphytic Diatoms from Terra Nova Bay and Cape Evans (Ross Sea, Antarctica) - A Synthesis and Final Conclusions. PLOS ONE.

[B9043090] Margules C. R., Pressey R. L. (2000). Systematic conservation planning. Nature.

[B11068601] Mashamaite Lefentse, Lebre Pedro H., Varliero Gilda, Maphosa Silindile, Ortiz Max, Hogg Ian D., Cowan Don A. (2023). Microbial diversity in Antarctic Dry Valley soils across an altitudinal gradient. Frontiers in Microbiology.

[B10820796] Maturana Claudia S., Biersma Elisabeth M., Díaz Angie, González-Wevar Claudio, Contador Tamara, Convey Peter, Jackson Jennifer A., Poulin Elie (2022). Survivors and colonizers: Contrasting biogeographic histories reconciled in the Antarctic freshwater copepod *Boeckellapoppei*. Frontiers in Ecology and Evolution.

[B9140460] McMurdie Paul J., Holmes Susan (2013). phyloseq: An R package for reproducible interactive analysis and graphics of microbiome census data. PLOS One.

[B11068665] Meier Lars A., Krauze Patryk, Prater Isabel, Horn Fabian, Schaefer Carlos E. G. R., Scholten Thomas, Wagner Dirk, Mueller Carsten W., Kühn Peter (2019). Pedogenic and microbial interrelation in initial soils under semiarid climate on James Ross Island, Antarctic Peninsula region. Biogeosciences.

[B9043258] Meyer Carsten, Kreft Holger, Guralnick Robert, Jetz Walter (2015). Global priorities for an effective information basis of biodiversity distributions.. Nature communications.

[B9635423] Mikucki J A, Lee P A, Ghosh D, Purcell A M, Mitchell A C, Mankoff K D, Fisher A T, Tulaczyk S, Carter S, Siegfried M R, Fricker H A, Hodson T, Coenen J, Powell R, Scherer R, Vick-Majors T, Achberger A A, Christner B C, Tranter M (2016). Subglacial Lake Whillans microbial biogeochemistry: a synthesis of current knowledge.. Philosophical transactions. Series A, Mathematical, Physical, and Engineering Sciences.

[B9714563] Montero Estrella, González Luis Miguel, Chaparro Alberto, Benzal Jesús, Bertellotti Marcelo, Masero José A, Colominas-Ciuró Roger, Vidal Virginia, Barbosa Andrés (2016). First record of *Babesia* sp. in Antarctic penguins.. Ticks and Tick-borne Diseases.

[B9635121] Ochyra R., Smith R. I. Lewis, Bednarek-Ochyra H. (2008). The illustrated moss flora of Antarctica.

[B11068644] Ortiz Maximiliano, Leung Pok Man, Shelley Guy, Jirapanjawat Thanavit, Nauer Philipp A., Van Goethem Marc W., Bay Sean K., Islam Zahra F., Jordaan Karen, Vikram Surendra, Chown Steven L., Hogg Ian D., Makhalanyane Thulani P., Grinter Rhys, Cowan Don A., Greening Chris (2021). Multiple energy sources and metabolic strategies sustain microbial diversity in Antarctic desert soils. Proceedings of the National Academy of Sciences.

[B9140146] Overmann Jörg, Huang Sixing, Nübel Ulrich, Hahnke Richard L., Tindall Brian J. (2019). Relevance of phenotypic information for the taxonomy of not-yet-cultured microorganisms. Systematic and Applied Microbiology.

[B9635073] Øvstedal D. O., Lewis Smith R. I. (2001). Lichens of Antarctica and South Georgia. A guide to their identification and ecology.

[B9140479] Pagès H., Aboyoun P., Gentleman R., DebRoy S. (2021). Biostrings: Efficient manipulation of biological strings. https://github.com/Bioconductor/Biostrings.

[B9382280] Park Kyung-Min, Jung Jae-Ho, Kim Jeong-Hoon, Min Gi-Sik, Kim Sanghee (2020). Morphology, morphogenesis, and molecular phylogeny of a new freshwater ciliate, *Gonostomumjangbogoensis* n. sp. (Ciliophora, Hypotricha), from Victoria Land, Antarctica. European Journal of Protistology.

[B10816310] Peat Helen J. (1998). The Antarctic Plant Database: a specimen and literature based information system. TAXON.

[B9382257] Peat Helen J., Clarke Andrew, Convey Pete (2007). Diversity and biogeography of the Antarctic flora. Journal of Biogeography.

[B9143128] Pertierra Luis R., Convey Peter, Martinez Pablo Ariel, Tejedo Pablo, Benayas Javier, Olalla-Tárraga Miguel Ángel (2022). Can classic biological invasion hypotheses be applied to reported cases of non-native terrestrial species in the Maritime Antarctic?. Antarctic Science.

[B9139214] Pertierra L. R., Santos-Martin F., Hughes K. A., Avila C., Caceres J. O., De Filippo D., Gonzalez S., Grant S. M., Lynch H., Marina-Montes C., Quesada A., Tejedo P., Tin T., Benayas J. (2021). Ecosystem services in Antarctica: Global assessment of the current state, future challenges and managing opportunities. Ecosystem Services.

[B9043034] Phillips Laura M., Leihy Rachel I., Chown Steven L. (2022). Improving species-based area protection in Antarctica. Conservation Biology.

[B11068744] Picazo Antonio, Villaescusa Juan Antonio, Rochera Carlos, Miralles-Lorenzo Javier, Quesada Antonio, Camacho Antonio (2021). Functional Metabolic Diversity of Bacterioplankton in Maritime Antarctic Lakes. Microorganisms.

[B9143063] Pyle Richard L., Barik Saroj K., Christidis Les, Conix Stijn, Costello Mark John, van Dijk Peter Paul, Garnett Stephen T., Hobern Donald, Kirk Paul M., Lien Aaron M., Orrell Thomas M., Remsen David, Thomson Scott A., Wambiji Nina, Zachos Frank E., Zhang Zhi-Qiang, Thiele Kevin R. (2021). Towards a global list of accepted species V. The devil is in the detail. Organisms Diversity & Evolution.

[B9140217] Qiang-long Zhu, Shi Liu, Peng Gao, Fei-shi Luan (2014). High-throughput sequencing technology and its application. Journal of Northeast Agricultural University (English Edition).

[B9140378] Quast Christian, Pruesse Elmar, Yilmaz Pelin, Gerken Jan, Schweer Timmy, Yarza Pablo, Peplies Jörg, Glöckner Frank Oliver (2012). The SILVA ribosomal RNA gene database project: improved data processing and web-based tools. Nucleic Acids Research.

[B11068755] Ramoneda Josep, Hawes Ian, Pascual-García Alberto, J. Mackey Tyler, Y. Sumner Dawn, D. Jungblut Anne (2021). Importance of environmental factors over habitat connectivity in shaping bacterial communities in microbial mats and bacterioplankton in an Antarctic freshwater system. FEMS Microbiology Ecology.

[B9140451] Team R Core (2021). R: A language and environment for statistical computing. R Foundation for Statistical Computing, Vienna..

[B9635338] Robert Vincent, Vu Duong, Amor Ammar Ben Hadj, van de Wiele Nathalie, Brouwer Carlo, Jabas Bernard, Szoke Szaniszlo, Dridi Ahmed, Triki Maher, Ben Daoud Samy, Chouchen Oussema, Vaas Lea, de Cock Arthur, Stalpers Joost A, Stalpers Dora, Verkley Gerard J M, Groenewald Marizeth, Dos Santos Felipe Borges, Stegehuis Gerrit, Li Wei, Wu Linhuan, Zhang Run, Ma Juncai, Zhou Miaomiao, Gorjón Sergio Pérez, Eurwilaichitr Lily, Ingsriswang Supawadee, Hansen Karen, Schoch Conrad, Robbertse Barbara, Irinyi Laszlo, Meyer Wieland, Cardinali Gianluigi, Hawksworth David L, Taylor John W, Crous Pedro W (2013). MycoBank gearing up for new horizons.. IMA Fungus.

[B9634884] Rodriguez P., Rico E. (2008). A new freshwater oligochaete species (Clitellata: Enchytraeidae) from Livingston Island, Antarctica. Polar Biology.

[B9634838] Rosa Luiz Henrique, da Silva Thamar Holanda, Ogaki Mayara Baptistucci, Pinto Otávio Henrique Bezerra, Stech Michael, Convey Peter, Carvalho-Silva Micheline, Rosa Carlos Augusto, Câmara Paulo E A S (2020). DNA metabarcoding uncovers fungal diversity in soils of protected and non-protected areas on Deception Island, Antarctica.. Scientific Reports.

[B9713332] Roskov Yuri, Ower G., Orrell Thomas M., Nicolson David, Bailly Nicolas, Kirk Paul Michael, Bourgoin Thierry, DeWalt R. Edward, Decock Wim, van Nieukerken Erik J., Zarucchi James Lee, Penev Lyubomir (2020). Species 2000 & ITIS catalogue of life, 2019 annual checklist. Catalogue of Life.

[B9043423] Ruggiero Michael A., Gordon Dennis P., Orrell Thomas M., Bailly Nicolas, Bourgoin Thierry, Brusca Richard C., Cavalier-Smith Thomas, Guiry Michael D., Kirk Paul M. (2015). A higher level classification of all living organisms. PLOS One.

[B9635764] Ruprecht Ulrike, Georg Brunauer, Christian Printzen (2012). Genetic diversity of photobionts in Antarctic lecideoid lichens from an ecological view point. The Lichenologist.

[B10820689] Saucède Thomas, Eléaume Marc, Jossart Quentin, Moreau Camille, Downey Rachel, Bax Narissa, Sands Chester, Mercado Borja, Gallut Cyril, Vignes-Lebbe Régine (2020). Taxonomy 2.0: computer-aided identification tools to assist Antarctic biologists in the field and in the laboratory. Antarctic Science.

[B11068850] Saxton Matthew A., Samarkin Vladimir A., Madigan Michael T., Bowles Marshall W., Sattley William Matthew, Schutte Charles A., Joye Samantha B. (2021). Sulfate reduction and methanogenesis in the hypersaline deep waters and sediments of a perennially ice‐covered lake. Limnology and Oceanography.

[B9140391] Schoch Conrad L, Ciufo Stacy, Domrachev Mikhail, Hotton Carol L, Kannan Sivakumar, Khovanskaya Rogneda, Leipe Detlef, Mcveigh Richard, O’Neill Kathleen, Robbertse Barbara, Sharma Shobha, Soussov Vladimir, Sullivan John P, Sun Lu, Turner Seán, Karsch-Mizrachi Ilene (2020). NCBI Taxonomy: a comprehensive update on curation, resources and tools. Database.

[B11068731] Severgnini Marco, Canini Fabiana, Consolandi Clarissa, Camboni Tania, Paolo D'Acqui Luigi, Mascalchi Cristina, Ventura Stefano, Zucconi Laura (2021). Highly differentiated soil bacterial communities in Victoria Land macro-areas (Antarctica). FEMS Microbiology Ecology.

[B9042957] Shaw Justine D., Terauds Aleks, Riddle Martin J., Possingham Hugh P., Chown Steven L. (2014). Antarctica’s protected areas are inadequate, unrepresentative, and at risk. PLOS Biology.

[B9714633] Short K A, Sands C J, McInnes S J, Pisani D, Stevens M I, Convey P (2022). An ancient, Antarctic-specific species complex: large divergences between multiple Antarctic lineages of the tardigrade genus *Mesobiotus*.. Molecular Phylogenetics and Evolution.

[B9139186] Siegert Martin, Atkinson Angus, Banwell Alison, Brandon Mark, Convey Peter, Davies Bethan, Downie Rod, Edwards Tamsin, Hubbard Bryn, Marshall Gareth, Rogelj Joeri, Rumble Jane, Stroeve Julienne, Vaughan David (2019). The Antarctic Peninsula under a 1.5°C global warming scenario. Frontiers in Environmental Science.

[B11068622] Solon Adam J., Mastrangelo Claire, Vimercati Lara, Sommers Pacifica, Darcy John L., Gendron Eli M. S., Porazinska Dorota L., Schmidt S. K. (2021). Gullies and Moraines Are Islands of Biodiversity in an Arid, Mountain Landscape, Asgard Range, Antarctica. Frontiers in Microbiology.

[B11068797] Sommers Pacifica, Darcy John L, Gendron Eli M S, Stanish Lee F, Bagshaw Elizabeth A, Porazinska Dorota L, Schmidt Steven K (2017). Diversity patterns of microbial eukaryotes mirror those of bacteria in Antarctic cryoconite holes. FEMS Microbiology Ecology.

[B9141209] Staebe K., Meiklejohn K. I., Singh S. M., Matcher G. F. (2019). Biogeography of soil bacterial populations in the Jutulsessen and Ahlmannryggen of Western Dronning Maud Land, Antarctica. Polar Biology.

[B9714603] Stevens M. I., D'Haese C. A. (2016). Morphologically tortured: taxonomic placement of an Antarctic springtail (Collembola: Isotomidae) misguided by morphology and ecology. Zoologica Scripta.

[B9141117] Taş Neslihan, de Jong Anniek EE, Li Yaoming, Trubl Gareth, Xue Yaxin, Dove Nicholas C (2021). Metagenomic tools in microbial ecology research. Current Opinion in Biotechnology.

[B9635782] Techow N M S M, O'Ryan C, Phillips R A, Gales R, Marin M, Patterson-Fraser D, Quintana F, Ritz M S, Thompson D R, Wanless R M, Weimerskirch H, Ryan P G (2009). Speciation and phylogeography of giant petrels *Macronectes*.. Molecular Phylogenetics and Evolution.

[B9042967] Terauds Aleks, Chown Steven L., Morgan Fraser, Helen J. Peat,, Watts David J., Keys Harry, Convey Peter, Bergstrom Dana M. (2012). Conservation biogeography of the Antarctic. Diversity and Distributions.

[B9042980] Terauds Aleks, Lee Jasmine R. (2016). Antarctic biogeography revisited: updating the Antarctic Conservation Biogeographic Regions. Diversity and Distributions.

[B9142983] Thiele Kevin R., Conix Stijn, Pyle Richard L., Barik Saroj K., Christidis Les, Costello Mark John, van Dijk Peter Paul, Kirk Paul, Lien Aaron, Thomson Scott A., Zachos Frank E., Zhang Zhi-Qiang, Garnett Stephen T. (2021). Towards a global list of accepted species I. Why taxonomists sometimes disagree, and why this matters. Organisms Diversity & Evolution.

[B9382270] Thompson Andrew R., Powell Gareth S., Adams Byron J. (2019). Provisional checklist of terrestrial heterotrophic protists from Antarctica. Antarctic Science.

[B9143001] Thomson Scott A., Thiele Kevin, Conix Stijn, Christidis Les, Costello Mark John, Hobern Donald, Nikolaeva Svetlana, Pyle Richard L., van Dijk Peter Paul, Weaver Haylee, Zachos Frank E., Zhang Zhi-Qiang, Garnett Stephen T. (2021). Towards a global list of accepted species II. Consequences of inadequate taxonomic list governance. Organisms Diversity & Evolution.

[B10816232] Treonis Amy M., Wall Diana H., Ross Virginia A. (1999). Invertebrate Biodiversity in Antarctic Dry Valley Soils and Sediments. Ecosystems.

[B9140237] Tringe Susannah G, Hugenholtz Philip (2008). A renaissance for the pioneering 16S rRNA gene. Current Opinion in Microbiology.

[B9141140] Tully Benjamin J., Graham Elaina D., Heidelberg John F. (2018). The reconstruction of 2,631 draft metagenome-assembled genomes from the global oceans. Scientific Data.

[B11068533] Tytgat Bjorn, Verleyen Elie, Sweetlove Maxime, D'hondt Sofie, Clercx Pia, Van Ranst Eric, Peeters Karolien, Roberts Stephen, Namsaraev Zorigto, Wilmotte Annick, Vyverman Wim, Willems Anne (2016). Bacterial community composition in relation to bedrock type and macrobiota in soils from the Sør Rondane Mountains, East Antarctica. FEMS Microbiology Ecology.

[B11068699] Varliero Gilda, Lebre Pedro H., Adams Byron, Chown Steven L., Convey Peter, Dennis Paul G., Fan Dandan, Ferrari Belinda, Frey Beat, Hogg Ian D., Hopkins David W., Kong Weidong, Makhalanyane Thulani, Matcher Gwynneth, Newsham Kevin K., Stevens Mark I., Weigh Katherine V., Cowan Don A. (2024). Biogeographic survey of soil bacterial communities across Antarctica. Microbiome.

[B9714653] Vecchi Matteo, Cesari Michele, Bertolani Roberto, Jönsson K. Ingemar, Rebecchi Lorena, Guidetti Roberto (2016). Integrative systematic studies on tardigrades from Antarctica identify new genera and new species within Macrobiotoidea and Echiniscoidea. Invertebrate Systematics.

[B9713303] Velázquez David, López-Bueno Alberto, Aguirre de Cárcer Daniel, de los Ríos Asunción, Alcamí Antonio, Quesada Antonio (2016). Ecosystem function decays by fungal outbreaks in Antarctic microbial mats.. Scientific Reports.

[B9043387] Verleyen Elie, Van de Vijver Bart, Tytgat Bjorn, Pinseel Eveline, Hodgson Dominic A., Kopalová Kateřina, Chown Steven L., Van Ranst Eric, Imura Satoshi, Kudoh Sakae, Van Nieuwenhuyze Wim, Sabbe Koen, Vyverman Wim (2021). Diatoms define a novel freshwater biogeography of the Antarctic. Ecography.

[B9635174] Vianna Juliana, Fernandes Flávia A. N., Frugone María José, Figueiró Henrique V., Pertierra Luis R., Noll Daly, Bi Ke, Wang-Claypool Cynthia Y., Lowther Andrew, Parker Patricia, Le Bohec Celine, Bonadonna Francesco, Wienecke Barbara, Pistorius Pierre, Steinfurth Antje, Burridge Christopher P., Dantas Gisele P. M., Poulin Elie, Simison W. Brian, Henderson Jim, Eizirik Eduardo, Nery Mariana F., Bowie Rauri C. K. (2020). Genome-wide analyses reveal drivers of penguin diversification. Dryad.

[B8812279] Wauchope Hannah S., Shaw Justine D., Terauds Aleks (2019). A snapshot of biodiversity protection in Antarctica. Nature Communications.

[B11068809] Webster-Brown Jenny G., Hawes Ian, Jungblut Anne D., Wood Susanna A., Christenson Hannah K. (2015). The effects of entombment on water chemistry and bacterial assemblages in closed cryoconite holes on Antarctic glaciers. FEMS Microbiology Ecology.

[B11068766] Weisleitner Klemens, Perras Alexandra, Moissl-Eichinger Christine, Andersen Dale T., Sattler Birgit (2019). Source Environments of the Microbiome in Perennially Ice-Covered Lake Untersee, Antarctica. Frontiers in Microbiology.

[B9043130] Whittaker Robert J., Araújo Miguel B., Jepson Paul, Ladle Richard J., Watson James E. M., Willis Katherine J. (2005). Conservation Biogeography: assessment and prospect. Diversity and Distributions.

[B10820787] Wilson R. (1997). A method for restraining penguins. Marine Ornithology.

[B9635211] Board WORMS Editorial World Register of Marine Species. https://www.marinespecies.org.

[B9141128] Yang Chao, Chowdhury Debajyoti, Zhang Zhenmiao, Cheung William K., Lu Aiping, Bian Zhaoxiang, Zhang Lu (2021). A review of computational tools for generating metagenome-assembled genomes from metagenomic sequencing data. Computational and Structural Biotechnology Journal.

[B9635600] Zablocki Olivier, van Zyl Lonnie, Adriaenssens Evelien M, Rubagotti Enrico, Tuffin Marla, Cary Stephen Craig, Cowan Don (2014). High-level diversity of tailed phages, eukaryote-associated viruses, and virophage-like elements in the metaviromes of antarctic soils.. Applied and Environmental Microbiology.

[B11068517] Zhang Eden, Thibaut Loïc M., Terauds Aleks, Raven Mark, Tanaka Mark M., van Dorst Josie, Wong Sin Yin, Crane Sally, Ferrari Belinda C. (2020). Lifting the veil on arid-to-hyperarid Antarctic soil microbiomes: a tale of two oases. Microbiome.

